# NF-κB signaling pathway in tumor microenvironment

**DOI:** 10.3389/fimmu.2024.1476030

**Published:** 2024-10-18

**Authors:** Yaning Cao, Yanan Yi, Chongxu Han, Bingwei Shi

**Affiliations:** ^1^ Department of Blood Transfusion, Changzhou Hospital of Traditional Chinese Medicine, Changzhou, Jiangsu, China; ^2^ Department of Laboratory Medicine, Northern Jiangsu People’s Hospital Affiliated to Yangzhou University, Yangzhou, Jiangsu, China

**Keywords:** NF-κB signaling pathway, inflammation, tumor microenvironment, cancer metabolism, tumor immunity

## Abstract

The genesis and progression of tumors are multifaceted processes influenced by genetic mutations within the tumor cells and the dynamic interplay with their surrounding milieu, which incessantly impacts the course of cancer. The tumor microenvironment (TME) is a complex and dynamic entity that encompasses not only the tumor cells but also an array of non-cancerous cells, signaling molecules, and the extracellular matrix. This intricate network is crucial in tumor progression, metastasis, and response to treatments. The TME is populated by diverse cell types, including immune cells, fibroblasts, endothelial cells, alongside cytokines and growth factors, all of which play roles in either suppressing or fostering tumor growth. Grasping the nuances of the interactions within the TME is vital for the advancement of targeted cancer therapies. Consequently, a thorough understanding of the alterations of TME and the identification of upstream regulatory targets have emerged as a research priority. NF-κB transcription factors, central to inflammation and innate immunity, are increasingly recognized for their significant role in cancer onset and progression. This review emphasizes the crucial influence of the NF-κB signaling pathway within the TME, underscoring its roles in the development and advancement of cancer. By examining the interactions between NF-κB and various components of the TME, targeting the NF-κB pathway appears as a promising cancer treatment approach.

## 1 Introduction

The occurrence and development of tumors, from metastasis to treatment resistance, result from the mutual interaction between cancer cells and the tumor microenvironment (TME). The TME, a complex and dynamic system, primarily comprises tumor cells, adjacent immune and inflammatory cells, tumor-related fibroblasts, surrounding stromal tissues, microvessels, and various cytokines and chemokines (1). Tumor formation is a complex and detailed biological process where cancer cells and their surrounding TME continuously interact and regulate tumor progression. The mutations within tumor cells, abnormalities in biological traits, persistent inflammation around the tumor and the remodeling of the extracellular Matrix (ECM) represent several closely related biological processes (2, 3). Notably, the components of the TME contribute to various cancer hallmarks, thereby being acknowledged as potential targets for cancer therapy. The TME encompasses both stromal and non-cellular components. Stromal cells consist of immune cells (lymphoid cells, tumor-associated macrophages (TAMs), dendritic cells (DCs), cancer-associated fibroblasts (CAFs), endothelial cells, and pericytes). Non-cellular components include the Extracellular Matrix (ECM), extracellular vesicles (EVs) or exosomes, and the microbiome (3). Tumor-associated stromal cells provide physical support to cancer cells and secrete growth factors, cytokines, chemokines, and ECM proteins that promote tumor growth.

The NF-κB signaling pathway plays a critical role in these processes, which is well-known for its role in regulating immune and inflammatory responses, and its activity is intricately linked with various components of the TME. Persistent activation of NF-κB within the TME can promote chronic inflammation, which in turn supports tumor growth, survival, and metastasis. Given its central role in inflammatory signaling, the relationship between the NF-κB signaling pathway and the TME has garnered considerable interest. Understanding this link is essential for developing targeted therapies aimed at disrupting the pro-tumorigenic interactions within the TME.

## 2 NF-κB signaling pathway in glance

The nuclear transcription factor NF-κB, recognized for its ability to bind to the enhancer element of the Kappa light chain gene in B cells, plays a pivotal role in regulating innate and adaptive immune responses. Its influence extends across a wide array of biological functions, including cell proliferation, apoptosis, angiogenesis, and tumor metastasis (4). The NF-κB signaling pathway is divided into canonical and non-canonical pathways (5). The canonical NF-κB pathway facilitates the activation of transcription factors such as NF-κB1 (p50), Rel- A (p65), and c-Rel, integral members of the classical NF-κB family. This pathway is quickly and transiently triggered by various extracellular stimuli through receptors like cytokine receptors, pattern recognition receptors (PRRs), tumor necrosis factor receptors (TNFRs), G protein-coupled receptors (GPCRs), T cell receptors (TCRs), and B cell receptors (BCRs) (6).

Conversely, the non-canonical pathway is specifically activated by a selective group of TNF-family cytokines, including lymphotoxin (LT), receptor activator of NF-κB ligand (RANKL), CD40 ligand (CD40L), and B cell activating factor of the TNF family (BAFF/TNFSF13B) (7). Unlike the canonical pathway, the non-canonical route gradually induces the activation of IKK1 and NF-κB inducing kinase (NIK), ultimately influencing the interaction between p52 and Rel-B (8).

In an inactive state, NF-κB is sequestered in the cytoplasm by the IκB-α proteins, which inhibit NF-κB signaling. In respond to inflammatory stimulation, IκB kinases (IKKs) are activated, phosphorylating IκB-α and leading to its ubiquitination by the E3 ubiquitin ligase β-TRCP. This process results in the degradation of IκB-α, freeing NF-κB to migrate to the nucleus and modulate the expression of genes such as tumor necrosis factors-α (TNF-α),interleukin-1(IL-1), and IL-2 (9, 10).

**Figure 1:**
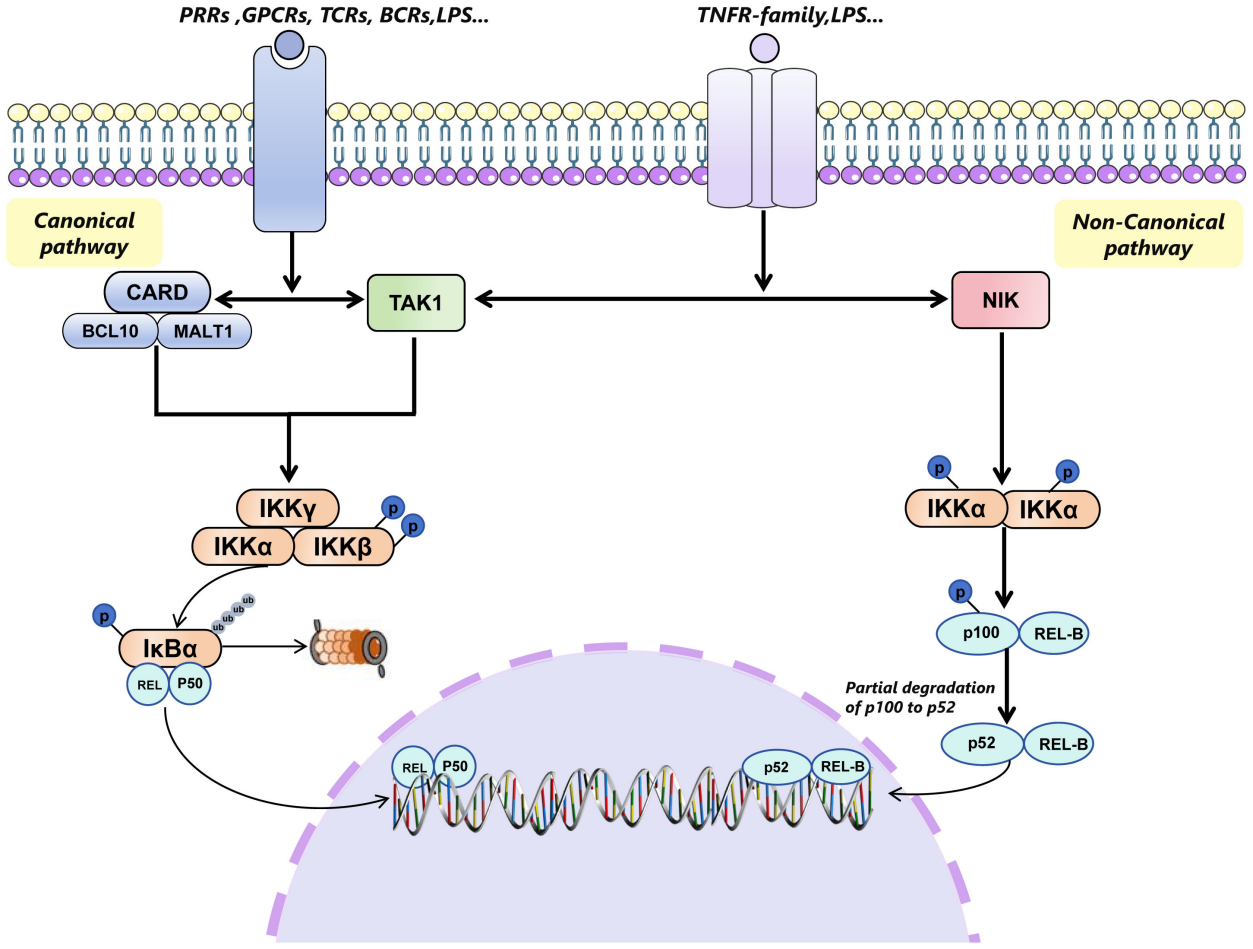
Canonical pathway and non-canonical pathway of NF-κB signaling pathway. The canonical NF-κB signaling pathway is initiated by various extracellular stimuli through different receptors, including TLRs, PRRs, TNFRs, GPCRs, TCRs, and BCRs. This activation occurs through the CARD-BCL10-MALT1 (CBM) complex and TAK1, leading to the phosphorylation and activation of the IKK complex (comprising IKKγ(NEMO), IKKα, and IKKβ) specifically through the phosphorylation of IKKβ. The activated IKK complex then phosphorylates members of the inhibitor of κB (IκB) family, such as IκBα, targeting them for ubiquitin (Ub)-dependent degradation in the proteasome. This degradation process releases the Rel-A/p50 or c-Rel/p50 or p50/p50(only Rel-A/50 is shown in **Figure 1**), which translocates to the nucleus to regulate gene transcription. In contrast, the non-canonical pathway is activated by the members of TNFR superfamily (such as CD40, LTβR and RANK), which specifically engage NIK. NIK then phosphorylates IKKα, leading to the phosphorylation of p100 on its carboxy-terminal serine residues. The phosphorylated p100 undergoes partial degradation to p52, which then associates with REL-B and translocates to the nucleus to participate in transcription events. TLRs, TLR toll-like receptor; PRPs, pattern recognition receptors; TNFRs, tumor necrosis factor receptors; GPCRs, G protein-coupled receptors; TCRs, T cell receptors; BCRs, B cell receptors; TANK1, TGF-β-activated kinase 1; LTβR, LTB lymphotoxin β;RANK, Receptor Activator of NF-κB; NIK, NF-κB-inducing kinase.

Notably, IKKs consists of two catalytic subunits, IKKα (IKK1) and IKKβ (IKK2), and a regulatory subunit, IKKγ (NEMO). IKKβ is essential for classical NF-κB activation, while IKKα primarily facilitates non-classical NF-κB activation (11) (**Figure 1**).

## 3 NF-κB-mediated shaping of the tumor immunity

### 3.1 NK cells

Natural killer (NK) cells, key effector lymphocytes of the innate immune system, play a crucial role in the suppression of various neoplasms through the secretion of cytolytic mediators such as perforin and granzyme B (12). It was also reported that NK cells turn cancer cells to death directly via perforin and granzyme B, which endorses them to be a valuable tool in cancer treatment (13, 14).

The NF-κB pathway is a key regulator of perforin expression, as demonstrated by Jun Zhou et al. in 2002 (15). Subsequent research by Chunjian Huang et al. in 2006 unveiled a novel NF-κB binding site instrumental in modulating human granzyme B gene transcription in a IL-2 signaling-dependent way (16). Recent studies have demonstrated that, in a head and neck squamous cell carcinoma (HNSCC) model, the ablation of the CHMP2A gene precipitates NF-κB activation in neoplastic cells, culminating in augmented chemokine secretion that facilitates NK cells migration towards the tumor microenvironment (17). In the breast cancer model, the protein Morgana—ubiquitously expressed and pivotal for embryonic development as well as tumorigenesis—has been shown to amplify NF-κB activation, thereby enhancing the recruitment of NK cells and other immune cells to the TME (18, 19).

### 3.2 T cells

It is well established that NF-κB is essential in the differentiation and function of lymphocytes (20). During T cell development, NF-κB is activated by TCR-peptide-MHC complexes via CARMA1-Bcl1-Malt1(CBM) complex(activated by PKCθ), controlling both the positive and negative selction (20). In contrast to its role in normal lymphocyte development, T cells and B cells recruited to the tumor microenvironment (TME) can either promote or inhibit tumor growth, depending on their specific functions. CD8^+^ T cells, namely cytotoxic T cells, kill immunogen by secreting cytotoxic cytokines, including IFN-γ and Granzyme (21). Research has shown that expressing a constitutively active form of IKKβ in T cells enhances NF-κB activity, promoting an anti-tumor response that depends on IFN-γ-producing tumor-specific CD8^+^ T cells (22), highlighting the therapeutic potential of acceleration the IKKβ/NF-κB axis to augment antitumor immunity. A20, a ubiquitin-modifying enzyme encoded by the TNFAIP3 gene, is a critical negative regulator of NF-κB. Giordano et al. demonstrated that deleting A20 in CD8^+^ T cells enhances their cytotoxic function in an NF-κB-dependent manner, illustrating NF-κB’s positive role in anti-tumor activity (23). Analysis of patient tumor samples revealed that overall lung tumor NF-κB activity fosters T cell infiltration, attributable to the downregulation of chemokine ligand 20(CCL2)expression linked with tumor growth (24). This inspires us that the maintenance of appropriate NF-κB activity in CD8 T cells is of great importance for antitumor immunity, offering potential clinical applications. Another evidence of a pro-tumoral effect of NF-κB is that overexpression of NIK significantly reduced tumor size and extended survival in MC38 colorectal cancer and B16F10 melanoma models. This was accompanied by an increase in tumor-infiltrating CD4^+^ and CD8^+^ T cells, including IFN-γ-producing CD8^+^ T cells (25).

Compared to CD8^+^T cells, the indirect impact of CD4^+^ in TME has aroused increasing attention. Upon stimulated by antigen-peptide-MHC class II complex, CD4^+^ T cells undergo a multi-step differentiation process to give rise to different CD4^+^ helper T cell subsets, including Th1, Th2, Th17, Th9 and regulatory T cells (Tregs). In TME, CD4^+^T cells are recruited to cancer cells by various chemotactic factors and adhesion molecules. Depending on the expression of MHC class II molecules on the tumor cell’s surface, CD4 T cells will adopt different strategies to try to kill the cancer cells. When cancer cells highly express MHC II molecules, antigen-presenting cells (APCs) present cancer cells to CD8^+^ T cells. In such cases, CD4^+^ T cells mainly play an indirect function in helping effector T cells kill cancer cells. However, when tumor cells lack MHC class II molecules, CD4^+^ T cells will play a role by secreting various cytokines or assisting other immune cells (26). Conventional CD4^+^Foxp3^−^ T cells (Tconv) is considered as a critical role in the effect of anti-tumor responses. And the function in various diseases is relay on the differentiation of Tconv, which is the result from the stimulation of antigen or drive of cytokine microenvironment. Guilhem Lalle found that different NF-κB subunits has different functions relevant to the context of autoimmunity. In an active murine model experimental autoimmune encephalomyelitis (EAE) model, Rel-A is indispensable for the transition from Tconv to Th17 and thereby protects against inflammation (27). In contrast, in B16-OVA melanoma tumor bearing model, c-Rel rather than Rel-A, was essential for the control of tumor growth and enhancement of anti-PD-1 treatment by impacting CD4^+^ Tconv (27). Hyunju Oh and Yenkel Grinberg-Bleyer identified c-Rel as crucial for thymic regulatory T cell development, while p65 is vital for maintaining mature Treg identity and immune tolerance (28). Activated Tregs (aTregs), a subset of Tregs, are known to migrate to inflammatory tissues and the tumor microenvironment, where they act as effective inhibitors of anti-tumor activity. The same research team ulteriorly demonstrated deletion of NF-κB subunit c-Rel in tumor-bearing mice displays a profound decrease of aTregs in TME and ultimately suppresses melanoma growth by boosting the anti-tumor response (29). The different effects of c-Rel on different T cell subsets and the partially redundant effects of different NF-κB isoforms suggest that the integration and construction of the tumor immune microenvironment by NF-κB is complex and requires a nuanced approach to balance complex processes when targeting NF-κB as a tumor therapy.

The CBM (CARMA1-BCL10-MALT1) complex acts as a nexus for the activation of canonical NF-κB signaling following extracellular antigen stimulation through TCR and BCR (30). The loss of *Carma1* in a fraction of Tregs is sufficient to augment antitumor activity and control of tumor size. Importantly, the deletion of CARMA1 or inhibition of MALT1 reverses the insensitivity to the PD-1 blockade therapy via inducing IFN-γ-secretion in TME. The CBM complex/NF-κB pathway activation also triggers the expression of hypoxia-inducible factor-1 (HIF-1α) and IL-6, which initiate the formation of the TME. These molecules further drive Treg proliferation and migration through the MAPK/CDK4/6/Rb and STAT3/SIAH2/P27 pathways, reinforcing the immunosuppressive nature of the TME and accelerating tumor progression (31).

However, in the process of infection and cancer, the cytotoxicity function of CD8^+^ T undergoes a slow decay which is termed as T cells exhaustion. This exhaustion, driven by factors in the TME, results in a weakened immune response and is marked by the expression of immune checkpoint proteins. One of the primary strategies employed by tumors to evade immune surveillance is through the expression of immunosuppressive receptors such as Programmed Cell Death-1(PD-1) and cytotoxic T lymphocyte-associated antigen-4(CTLA-4) on effector T cells by their ligands present in the different cell types in TME (32), which leads to the blockade of effector function of normal T cells. Strategies for targeting T and B lymphocytes focus on blocking immune checkpoints, such as CTLA-4 and the PD-1/PD-L1 axis (33). The conditional CD28 knockout model proved that CD28 is needed for CD8^+^T cells proliferation after PD-1 therapy. The reversion of exhaustion of T cells is CD28-dependent and ultimately enhances the sensitivity of cancer cells to checkpoint blockade.TCR-CD28 co-stimulation is known to activate the CBM complex/NF-κB pathway in T cells, indicating that NF-κB could have a role in immune checkpoint therapy, although this needs further investigation (34). In addition to lymphoid cells, myeloid cells also express checkpoints that used to be targeted in cancer therapy. So some metabolic research has already tried to explain this by the regulation of NF-κB for metabolic reprogramming between oxidative phosphorylation (OXPHOS) and glycolysis in MDSC, which alongside with the change of checkpoints such as PD-1 in MDSC. This process eventually influences anti-immunity such as CD8^+^T cells responses (35).

### 3.3 B cells

Compared to T cell, the role of B cells within the TME is multifaceted and presents significant challenges. It has been established that NF-κB signaling pathway is essential in the maturation of B cells. Thomas Pohl et al. found the combined deletion of c-Rel and NF-κB1(p105/p50) displays severe humoral immunity due to profound B cell activation defects (36). In TME, the cancer local environment exhibits an increased presence of switched memory B cells and antibody-secreting B cells, suggesting a potential regulatory role of B cells in tumor progression via the modulation of cytokines, including inflammatory factors (37). Under CXCL13 stimulation, the NF-κB signaling pathway in B cells is activated, leading to the secretion of lymphotoxin (LT), a cytokine that triggers an IKKα–Polycomb complex protein BMI1 pathway. This pathway promotes the metastasis of prostate cancer stem cells by increasing leukocyte infiltration (38).

### 3.4 TAMs

Macrophages, particularly tumor-associated macrophages (TAMs), are the most abundant and critical cellular components of the TME, orchestrating various aspects of tumor immunity. M1 macrophages, activated through classical pathways, promote antitumor immunity and secrete pro-inflammatory cytokines such as TNF-α, IL-1β, and IL-6. In contrast, alternatively, activated M2 macrophages are linked to pro-tumorigenic activities and anti-inflammatory functions, including tissue repair, angiogenesis, and immunosuppression mediated by IL-10 and transforming growth factor-beta(TGF-β) (39, 40). The classical activation of macrophages, initiated by interferon-gamma (IFN-γ), differs from the alternative activation pathway, which is induced by glucocorticoids, IL-4, IL-13, and IL-10, leading to an immunosuppressive profile (41). Macrophages embody a paradoxical characteristic, capable of both pro-tumorigenic and anti-tumorigenic activities, reflecting their high plasticity and heterogeneity within the TME. In many cancers, including breast, cervical, and prostate cancers, TAMs, displaying generally M2 phenotype and contributing to tumor progression, and their high infiltration is often associated with poor patient prognosis (42). On the other hand, some reports also proved that the high density of TAMs infiltration in the tumor front has a positive relationship with the prognosis of CRC patients (40). Similarly, in colorectal cancer, macrophage in the TME ultimately polarized toward the anti-tumorgenesis phenotype(M1 phenotype) due to the attraction of T cells and the production of pro-inflammatory cytokines and chemokines (43). Taken together, these finding shows the complicated role of TAMs in cancer, which may be credited to the heterogeneity of different local tumor circumstances. As a result, researchers are increasingly focused on identifying and distinguishing TAM subtypes in the TME to better guide treatment strategies and predict patient prognosis by using innovative technologies.

Various signaling have proved to shape the phenotype of TAM, including STAT3, MAPK, IL12-R, Notch signaling pathway and Sema3A/Neuropilin-1 signaling axis et al (39, 40, 43). Among these signaling pathways, NF-κB is a regulating hub to balance between anti-tumorigenic and the pro-tumorigenic functions of TAMs. In 2008, Thorsten Hagemann and colleagues demonstrated that the depletion of IKK-β activity within TAMs can revert their tumor-promoting phenotype to antitumor M1 phenotype, indicating that reprogramming TAMs by target IKK-β target to eliminate tumor cells (44). Another intriguing study showed targeting NF-κB to induce an M1-like macrophage phenotype significantly reduced peritoneal metastasis of colon tumor *in vivo*. This effect was attributed to increased frequencies of activated CD4^+^ and CD8^+^ T cells and reduced angiogenesis (45). The research mentioned above demonstrated that NF-κB is an important driver of the M2-phenotype of TAMs in the tumor islet and eventually promotes the tumor progression via alteration of the inflammation or T cell infiltration in TME. Furthermore, overexpression of the p50 subunit of NF-κB is responsible for the diminished effect of M1-type macrophages *in vivo* and *in vitro*, which eventually changes the inflammation environment of TME (46). In addition to the inflammatory environment, the regulation of angiogenic factors and chemokines by NF-κB in macrophages represents a critical area of focus in cancer research. Vascular Endothelial Growth Factor (VEGF), as a key component of vasculogenesis and angiogenesis during development and physiological homeostasis, has been proven to be upregulated by the inflammatory mediator lipopolysaccharide (LPS) and by engagement of CD40 by CD40 ligand (CD40L), which is dependent the activation of NF-κB signaling (47). These findings describe that a series of cytokines and chemokines regulated by NF-κB in macrophages in the tumor microenvironment affects the inflammatory environment and pro-angiogenic environment in the tumor islet, thereby affecting the effect of the microenvironment on tumor progression in a different aspect.

However, contradictory results emerged in recent seminal projects. Cycling hypoxia(CyH) is a statement of intermittent hypoxia and particularly exists in TAMs. In monocytes, CyH enhances the pro-inflammatory phenotype of M1 macrophages evidenced by increased production of pro-inflammatory cytokines and expression of pro-inflammatory genes by activation of JNK/p65 signaling axis (48). It is an established fact that NF-κB activation in macrophages in the onset of inflammation is related to the expression of proinflammatory genes(e.g.TNF-α, IL-β), whereas NF-κB activation resolution phase is associated with the expression of anti-inflammatory genes(e.g. TGF-β1).In TME, similar to this phenomenon, TAMs driven by NF-κB during the beginning of the tumor tend to produce proinflammatory cytokines such as TNF-α and IL-β to suppress tumor growth. However, with the occurrence and development of tumors, NF-κB-mediated TAMs gradually turn to an anti-inflammatory property, which is manifested as immunosuppression (49, 50). VEGF also plays an indispensable role in the shaping of immunosuppressive microenvironment. On one hand, VEGF-A(a subunit of the VEGF superfamily) directly upregulates the expression of inhibitory receptors involved in T cell exhaustion including CTLA-4, PD-1 and Lag-3.On the other hand, targeting VEGF-A *in vitro* and *in vivo* reverts the inhibitory molecules which is associated with T exhaustion (51). The two coins inspire us that the relationship and crosstalk between T cells and macrophages regulated by NF-κB shape the characteristics of the tumor immune microenvironment. Furthermore, the duality and complexity of NF-κB-mediated TAMs enlighten us that a nuanced understanding of macrophage dynamics within the TME underscores the complexity of the immunological landscape in cancer. It offers insights into potential therapeutic strategies aimed at modulating macrophage function to combat tumor progression.

Of note, TAMs are shown to promote tumor development by maintaining an inflammatory microenvironment. Targeting TAMs’ inflammatory signaling pathways, such as the CSF1R pathway, is a current research focus (52). Additionally, TAMs express immune checkpoint ligands, suggesting that lymphocyte-targeting strategies could apply to TAMs as well (53). Targeting the NF-κB signaling pathway, a key chronic inflammatory pathway, has emerged as a significant research direction for cancer treatment.

### 3.5 DCs

Under physiological conditions, DCs are recognized as the most efficacious professional antigen-presenting cells (APCs), endowed with the capability to engulf, process, and present a diverse array of antigens, inclusive of tumor antigens, to antigen-specific naïve T cells. The growing evidences supports that the relationship between DCs and T cells, based on reciprocal signals exchanged during physical interactions, helps us to have a better understanding of the tension of immunity at the tumor site. The DCs in tumor islets function as shepherds in T cell anti-tumor effect cause DCs not only drive different signals into T cells which is essential in the development and maturation of effector T cell (54). Within the TME, however, DCs constitute a distinct subset of cells characterized by a unique phenotype and functional capacity, manifesting a dichotomous role that spans both pro-tumorigenic and anti-tumorigenic activities. It is well acknowledged that conventional DCs are generally divided into two subsets–cDC1s and cDC2s, which both develop in response to Flt3L from common myeloid progenitors (CMPs) and give rise to pre-cDC1s and pre-cDC2s (54). Recent findings underscored the dynamic nature of tumor-infiltrating dendritic cells (TIDCs), highlighting a pivotal transition from immunostimulatory to immunosuppressive functions as the neoplasm progresses (55). Barbara Maier identified a new cluster of DCs and named it with mature DCs enriched in immunoregulatory molecules (mregDCs), which express various immunoregulatory genes such as *Cd274, Pdcd1lg2and Cd200*. The expression of PD-L1 in mregDCs is induced by the receptor tyrosine kinase AXL with the negative control of IL-4 and positive regulation of IL-12 (56). This delicate regulator of DCs at the tumor site inspires the possibility of reversing the dysfunction and tolerance of DCs in TME to enhance the immunotherapy of cancer. Together these outcome not only delineates the intricate involvement of DCs within the neoplastic landscape but also elucidate a portion of the underlying complexity associated with their role in tumorigenesis.

Pertaining to the mechanistic underpinnings, it has been elucidated that the expression of PD-1 on DCs impedes NF-κB-dependent cytokine secretion through a mechanism contingent upon SHP-2 activation, which ultimately the dysfunction of DCs (57, 58). Yoshimura and colleagues proved that the expression of costimulatory molecules, MHC molecules, and production of various cytokines by DCs is downregulated by NF-κB activation, such as MHC class II-SIINFEKL complex, TNF-α, IL-6.In contrast, adhesion molecules are up-regulated after inhibiting the activity of NF-κB, indicating the additional effect of NF-κB on the interaction between DCs and T cells (57, 59). Through comparison between the control group and lung cancer patient sera and analysis of transcriptomic, the researcher found that the dysfunction of DCs in TME is controlled by the attenuating canonical NF-κB and STAT3 signaling, especially by reducing the antigen presentation ability of DCs (60). Of note, CCR7^+^DCs derived from cDC1s almost retain in tumor site and enhance anti-tumor immunity through mediating the expression of various chemokines and cytokines which are essential in the function of T cells and NK cells. The development of CCR7^+^DCs from cDC1s is dependent on transcription factor IFN regulatory factor 1 (IRF1), which is proved to be regulated by NF-κB in the maturation of tumor infiltrating cDC1s in melanoma model (61). This revelation contributes to a deeper understanding of the molecular pathways influencing DC functionality within the TME, shedding light on the nuanced interplay between immune surveillance and tumor evasion strategies. However, the general positive anti-tumor effect induced by NF-κB in DCs may be a contradiction to the negative effect of inflammation induced by NF-κB in the immune microenvironment such as TAMs and Treg, which shed light on tipping the nuanced balance in the therapy of cancer.

### 3.6 MDSCs

MDSCs represent a diverse assembly of pathologically activated, immature cellular entities playing a pivotal role in the orchestration of immunosuppressive networks. Characterized by their potent ability to inhibit T-cell mediated responses, MDSCs significantly contribute to the evasion of immune surveillance by malignant neoplasms (62). Emerging evidence underscores the role of MDSCs in tumor infiltration and promotion of angiogenesis, primarily through the secretion of matrix metallopeptidase 9 (MMP9) and their direct integration into the tumor endothelium, facilitating vasculogenesis (63). The deletion of c-Rel in melanoma and lymphoma mice model dramatically reduces the size and weight of the tumor. Compared to the control, the metabolism in Rel^-/-^ MDSCs was significantly reprogrammed with their mitochondrial respiratory parameters decreased and glycolysis enhanced. Notely, inhibiting c-Rel functions as a selective switch of anti-tumoral genes. All these data underscores the important role of c-Rel in the development of MDSCs that promote cancer (64). Furthermore, MDSCs are proved to be activated by the IL-1-induced NF-κB signaling pathway, which is thought to be one of mechanisms that fosters gastric inflammation and the proliferation of carcinoma cell (65). A crosstalk with STAT3 also regulates the functions of MDSCs. A research proved that the myeloid-related protein S100A9 induced by STAT3 enhances the accumulation and production of MDSCs in cancer (66). Another research proved that S100A8/A9-enhanced NADPH oxidase affects downstream NF-κB signaling pathway, which may play role in positive effect on MDSCs recruitment. This underlines the critical function of MDSCs in the modulation of tumor microenvironments via NF-κB, promoting both tumorigenesis and progression. The roles of different NF-κB subunits in the shaping of tumor immune microenvironment is concluded in **Table 1**.

**Table 1:** Roles of NF-κB in the shaping of tumor immune microenvironment.

Cell types	Function in Tumor Immunity	NF-κB pathway numbers involved	Effect of NF-κB to immunity	Reference
NK cells	Anti-tumor	NF-κB1/p50,RelA/p65	Enhancement of cytotoxicity	(15, 16, 19)
CD8^+^T cells	Anti-tumor	RelA/p65,IKKβ,NIK	Enhancement of cytotoxicity	(24, 25)
CD4^+^T cells	Anti-tumor	RelA/p65,c-Rel	Secretion of inflammatory cytokines	(26)
Tregs	Immunosuppression	RelA/p65,c-Rel	Development and maturation of Treg	(28, 29)
B cells	Anti-tumor	NF-κB1/p50, c-Rel	Development and function of B cells	(36)
M2 macrophages	Pro-tumor	IKK-β, IκB-α, NF-κB1/p50	Induction of tumoricidal activity; Activation of antitumor activity	(44–46)
M1 macrophages	Anti-tumor	RelA/p65	Enhancement of pro-inflammation	(48)
DCs	Anti-tumor	IKKβ	Development and maturation of DCs	(61)
MDSCs	Pro-tumor	c-Rel	Selective regulation of pro-tumoral genes	(64)

NK cells, Natural killer cells; NIK, NF-κB-inducing kinase; Tregs, regulatory T cells; DCs, Dentritic Cells; MDSCs, Myeloid-Derived Suppressor Cells.

## 4 Altering non-immune cells in the surroundings of tumor

### 4.1 CAFs

CAFs have been identified as pivotal constituents in the dialog between tumor cells and the TME, playing a central role in tumor progression. The fundamental contribution of CAFs to tumorigenesis, including tumor growth, invasion, and metastasis, is attributed to their capacity to modulate tumor-associated inflammation (67). CAFs contribute to breast and pancreatic cancer development by secreting cytokines like CXCL12 and CXCR4 and promoting metastasis through ECM remodeling (68). Targeting CAFs has potential in preventing tumor growth, with several drugs, including FAP, under clinical trial (69). NF-κB activation within the TME upregulates chemokines, which sustain the TME by recruiting immune and inflammatory cells, as well as progenitors of CAFs (70, 71). Studies have demonstrated that CAFs originating from skin tumors enhance macrophage recruitment, neovascularization, and tumor growth—effects that are abolished by the inhibition of NF-κB signaling (70).

IKKβ, a critical component of the IKK complex, is recognized not only for its essential role within this complex but also for its upstream regulation and pro-tumorigenic influence on NF-κB signaling. Contrary to previous findings, an interesting study revealed that IKKβ deficiency in CAFs promotes intestinal epithelial cell proliferation, inhibits tumor cell apoptosis, increases CD4^+^Foxp3^+^ regulatory T cell accumulation, and stimulates angiogenesis, thereby facilitating colonic tumor growth (72). As precursors to CAFs, mesenchymal cells also play a significant role in the acquisition of cancer characteristics through their interactions with adjacent epithelial and neoplastic cells, as well as other stromal cells. They contribute to the cancerous milieu by providing cytokines and chemokines, growth and survival factors, proangiogenic molecules, and enzymes for extracellular matrix remodeling. Koliaraki have demonstrated that specific deletion of IKKβ in intestinal mesenchymal cells(IMCs) *in vivo* results in reduced tumor incidence following exposure to azoxymethane(AOM) and dextran sodium sulfate(DSS) treatment, which is associated with diminished inflammatory cell infiltration and tissue damage in the initial stages of disease development (73, 74).

### 4.2 Epithelial cells/Endothelial cells

Epithelial cells, lining the surfaces of organs and body structures, can undergo significant transformations contributing to tumor development and the orchestration of the TME, angiogenesis, and metabolic reprogramming. Recent findings highlight the crucial role of NF-κB in modulating epithelial cell dynamics, primarily through its interactions with other signaling pathways. This interplay between NF-κB and various pathways is essential for regulating the phenotypic transformation of epithelial cells, laying the foundation for tumorigenesis. Notably, Schwitalla et al. demonstrated that NF-κB potentiates Wnt signaling, facilitating the dedifferentiation of epithelial non-stem cells into tumor-initiating cells. This process underscores the significance of NF-κB in the early stages of cancer development (75).

Additionally, IL-6, a downstream effector of NF-κB predominantly secreted by bone marrow-derived myeloid cells, plays a crucial role in this regulatory network. IL-6 activates the STAT3 pathway in both inflammatory and epithelial cells, leading to an increased nuclear presence of β-catenin. This key event in the pathogenesis of colorectal cancer highlights the interconnected roles of these signaling molecules in carcinogenesis (76).

The epithelial-to-mesenchymal transition (EMT) represents a critical shift where epithelial cells adopt mesenchymal characteristics, shedding their inherent epithelial traits (77). NF-κB influences this process by inducing transcription factors such as Twist and Snail, which are pivotal in orchestrating EMT (78, 79). In breast cancer cells, the involvement of NF-κB extends to mediating the expression of key EMT transcription factors, including Slug, Sip1, and Twist1,alongside NF-κB-dependent regulation of ZEB-1/ZFHX1A and ZEB-2/ZFHX1B, also known as Smad-interacting protein (78, 80). In addition, as an classic hallmarker of EMT,MMPs are zinc-dependent endopeptidases that can participate in proteolysis and can cleave several ECM components and non-ECM molecules (81). Identification of MMP-9 to barrier function in intestinal epithelial cell is not dependent on apoptosis and necrosis, but through the NF-κB mediating myosin light chain kinase (MLCK) protein and IL-6 expression, which gives explanation of the invasion of intestinal tumor (82). Conversely, suppression of p65 through siRNA result in the downregulating of MMP-9 in human oesophageal squamous cell cancer (ESCC) and inhibiting the proliferation and invasion ability ESCC (83). This intricate regulatory mechanism by NF-κB underscores its integral role in the modulation of EMT, further implicating its contribution to cancer progression.

**Figure 2:**
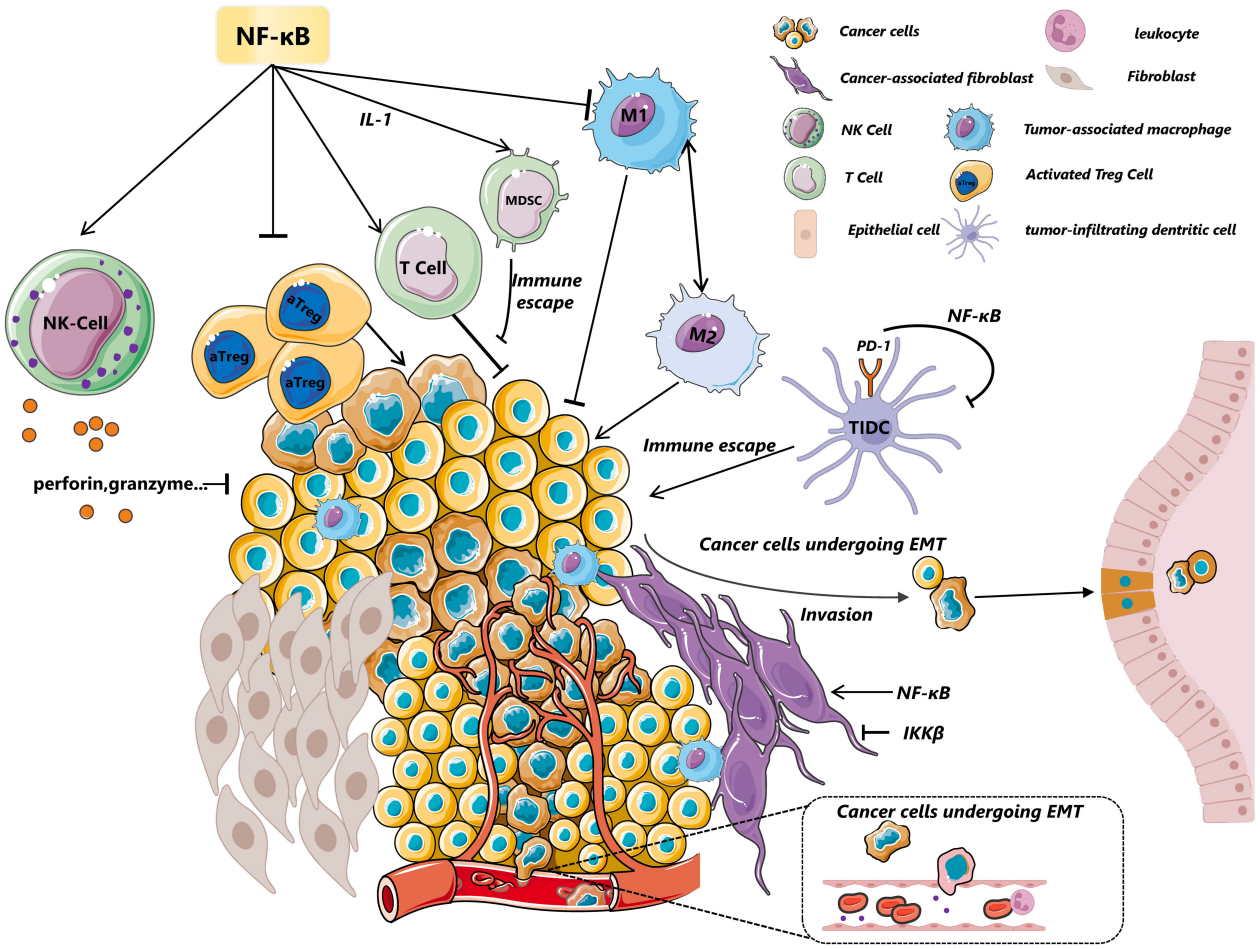
A glance of tumor microenvironment regulated by NF-κB. NF-κB modulates the dynamic state of the tumor microenvironment through its effects on various cells, including immune cells and myeloid cells, among others. In NK cells, the activation of the NF-κB pathway upregulates the expression of perforin and granzyme, crucial for inhibiting tumor growth and invasion. MDSCs are influenced by c-Rel-mediated IL-1 signaling, which suppresses T-cell-mediated responses and contributes to immune evasion. Furthermore, p50 or IKKβ drives TAMs towards a tumor-promoting M2 phenotype. In constrast, CyH enhance phenotype of M1 macrophages by activation of JNK/p65 signaling axis. The deletion of the NF-κB subunit c-Rel significantly reduces the generation and maintenance of activated regulatory T cells (aTregs), highlighting its essential role in immunosuppression. NF-κB(p65) also stimulates cancer-associated fibroblasts (CAFs) to enhance macrophage recruitment, neovascularization, and tumor growth via chemokines—a process that contrasts with the effects of IKKβ inhibition. IKKβ deficiency in CAFs promotes intestinal epithelial cell proliferation, inhibits tumor cell apoptosis, increases CD4+Foxp3+ regulatory T cell accumulation, and promote colonic tumor growth. Additionally, NF-κB promotes epithelial-mesenchymal transition (EMT) by inducing the transcription of factors like MMP-9,Twist and Snail, further illustrating its pivotal role in tumor progression and metastasis. CTCs, Circulating Tumor Cells; EMT, Epithelial–Mesenchymal Transition; MDSC, Myeloid-Derived Suppressor Cells; CAFs, Cancer-Associated Fibroblasts; TAMs, Tumor-Associated Macrophages; aTreg, Activated Treg cells; DCs, Dendritic Cells.

In contrast, endothelial cells(ECs) lining the inner vessel wall are in direct face with flowing blood, which is not only relevant for controlling blood fluidity and permeability and orchestrating tumor angiogenesis but also for regulating the antitumor immune response (84). Angiogenesis is thought to mainly include degradation of the endothelial basement membrane and ECM, and directed migration of endothelial cells into surrounding stroma in response to angiogenic stimuli. In ECs, MMPs as an important ECM protease are also crucial in the process of tumor cells’ transendothelial migration and acceleration of invasion and metastasis mainly through angiogenesis. Low fluid shear stress in human umbilical vein endothelial cells (HUVECs) greatly induced MMP-9 expression, which is interrupted by the inhibition of the activity of NF-κB (85). It is a fact that platelet-activating factor (PAF) accelerates angiogenesis by promoting various angiogenic factors in a manner dependent on NF-κB. Similarly, Hyun-Mi Ko found that overexpression of p65 induces the activity of MMP-9 luciferase and the mRNA expression of MMP-9, which plays a key role in PAF-induced angiogenesis (86). VEGF interacts with two primary receptor tyrosine kinases: VEGFR1 (fms-like tyrosine kinase, or Flt-1) and VEGFR2 (kinase insert domain receptor/fetal liver kinase-1, or KDR/flk-1) (87). These receptors, along with VEGF, are notably overexpressed in the endothelial cells of blood vessels associated with tumors (88). Research conducted by Fengyun Dong et al. revealed that DHA specifically reduces VEGFR2 expression and this process is connected by the NF-κB motif, suggesting that inhibition of NF-κB could serve as a viable strategy to mitigate tumor angiogenesis (89). Another important chemokine CXCL12, plays a key role in the communication of tumor cells and another component of TME and eventually affects tumor angiogenesis, proliferation and chemoresistance (90). In vascular endothelial cells, Madge et al. demonstrated that the non-canonical NF-κB pathway is crucial for the expression of CXCL12, emphasizing the pathway’s significance in mediating angiogenic signals (91). The NF-κB-inducing kinase (NIK) activates this non-canonical signaling route and is often found highly expressed in tumor tissues. Consistently, Noort et al. observed a notable reduction in the number of CXCL12-positive blood vessels which is dependent on non-canonical NF-κB in the tumor tissue of Nik-/-mice (92), highlighting non-canonical NF-κB components may serve as a focal point modulating vasculogenesis, angiogenesis and the attraction of immune cells (93). In response to various stimuli, endothelial cells secret and synthesize monocyte chemoattractant protein-1 (MCP-1), which is a major chemoattractant for monocytes, T lymphocytes, and basophils and changes the component and function of TME. NF-κB -like binding site and the AP-1 binding site are required for maximal induction of the human MCP-1 promoter by IL-1β, which suggests cytokines inducing MCP-1 in human endothelial cells is dependent on the coordination of NF-κB and AP-1 siganlings (94). A glance of tumor microenvironment regulated by NF-κB via different cell is reviewed in **Figure 2**.

### 4.3 Cancer cells

Many reviewers have concluded the tumorigenic process and draw a consensus that generally three phases of tumor: tumor initiation, tumor promotion and tumor progression. In the first stage of tumor, the DNA of tumor cell undergoes mutation and switches between oncogenes and/or the tumor-suppressor genes(e.g.KRAS and p53). The next step of tumorigenesis is the proliferation and growth of tumor cells which is mainly induced by various cytokines(e.g.IL-1, IL-6, TNF-α), which is concluded in the process of promotion. The final step is the invasion and metastasis of cancer cells, which is characterized by additional mutations and entitles the tumor cell with many malignant phenotypes (5).

It is well acknowledged that the activity of NF-κB within tumor cells is also associated with the dynamics of TME and changes the pace of progression of tumor. NF-κB is proven to be an activator of anti-apoptotic genes. In the process of tumor initiation, the activation of NF-κB in tumor cells mediates the epigenetic changes and instability of telomerase activity. For example, in diffuse large B cell lymphomas (DLBCL),p65 binds to the transcription start site (TSS) and regulates miRNA expression such as histone H3K27me3 and histone H3K4me3, which is associated with the progress of carcinoma (95). In human cancer cells, the reactivation of telomerase reactivation is one of the characteristics of cancer progression, which is attributed to the exhibit of a multitude of properties including increased proliferation, increased resistance to apoptosis and increased invasion. NF-κB transcriptionally upregulates telomerase levels, which binds to p65 and forms feedback to the enhancement of NF-κB -IL-6 axis (96). In the promotion of tumor, NF-κB is an essential switch of anti-apoptosis genes, which include cellular inhibitors of apoptosis (c-IAPs), caspase-8–c-FLIP (FLICE inhibitory protein), A1 (also known as Bfl1), TNFR-associated factor 1 (TRAF1) and TRAF2 (97). The anti-apoptotic properties in tumor cells to some extent endow these cells with the ability of unlimited proliferation and thus promote the occurrence and development of tumors. The inflammation induced by NF-κB is another important part of tumor promotor, which is mainly dependent on the immune cell in TME we have mentioned above. Last but not least, the invasion and metastasis of tumor cells can be regulated by the change of NF-κB. One example of this fact is the expression of matrix-degrading enzymes such as MMP-9 induced by NF-κB. In prostate carcinoma cells, inhibiting NF-κB activity resulted in the downregulation of MMP-9 mRNA, leading to decreased invasion of tumor. In addition, NF-κB signaling blockade inhibited *in vitro* and *in vivo* secretion of VEGF, IL-8, and MMP-9, and hence decreased neoplastic angiogenesis (98). MMP-9, upregulated in angiogenic islets and tumors, enhances the bioavailability of VEGF to its receptors, crucial for angiogenic switching and subsequent tumor growth (99). In breast cancer, the increasing of NF-κB activity leads to the higher expression of testes-specific protease 50 (TSP50) via regulating the secretion of MMP-9,which eventually promotes cell invasion and tumor metastasis (100). VEGF, an acknowledged most endothelial cell-specific angiogenic factor, is produced by various cell types, including endothelial cells(described in the former part) and cancer cells (47). In prostate cancer cells, bombesin(BBS) induced IκB degradation and activated NF-κB, resulting in increased IL-8 and VEGF mRNA expression and stronger migration of HUVECs *in vitro (* 101). Consistent with this finding, inhibition of NF-κB activity via IKK2 inhibitor attenuates the expression of VEGF‐A and HIF‐1α expression and some other inflammatory chemokines CCL2 and CXCL5 in TNF‐α‐stimulated HUVEC, thereby diminishing the infiltration of inflammatory cells into the corneal stroma (102). Urokinase‐type plasminogen activator receptor (uPAR) is displayed high level in in malignant tumors and is thought to be an attractive target for the therapy of many cancers. This interaction between uPAR and uPA initiates a proteolytic cascade that culminates in the degradation of the extracellular matrix (ECM), thereby facilitating the invasion and metastasis of malignant tumors (103). In a pioneering study conducted in 1999, Weixin Wang was the first to demonstrate that uPA is a downstream target gene activated by constitutively active Rel-A in human pancreatic tumor cells (104). This finding underscores the potential critical involvement of constitutive Rel-A activity in tumor progression, specifically in aspects of invasion and metastasis. Further extending our understanding of the molecular mechanisms underlying cancer metastasis, Sliva et al. revealed that Protein Kinase C (PKC) modulates cell motility by regulating uPA expression via the activation of transcription factors AP-1 and NF-κB in breast cancer. This body of work collectively highlights the intricate regulatory networks that govern tumor aggressiveness and offers valuable insights into potential therapeutic targets for combating cancer metastasis (105). The different effects of NF-κB on oncogenesis is concluded in **Table 2**.

**Table 2:** NF-κB regulates oncogenesis via different ways.

Ways		Cancer types	Target genes	Reference
Shaping of tumor immunity	NK cells	Breast carcinomas, Lung carcinomas, Urothelial carcinomas, Uasal-type NK/T-cell lymphoma	*Perforin↑*	(13, 15)
			*Granzyme B↑*	(14, 16)
	T cells	Lung carcinomas	*CCL20↓*	(24)
		Colon cancer	*HIF-α↑*	(31)
		Breast cancer	*IL-6↑*	(31)
	B cells	Prostate cancer	*LT↑*	(38)
	TAMs	Ovarian cancer	*IL-1↓*	(44)
		Ovarian carcinomas	*NOS↓*	(44)
	DCs	Ovarian carcinomas	*IL-6↑*	(59)
		Ovarian carcinomas	*TNF-α↑*	(59)
		Ovarian carcinomas	*IL-12↑*	(106)
		Melanoma, Ovarian carcinomas	IFN-γ	(61, 106)
Influencing stromal cells in TME	CAFs	Skin carcinomas	*CXCL1↑*	(70)
		Skin carcinomas	*CXCL12↑*	(70)
		Skin carcinomas	*IL-6↑*	(70)
		Skin carcinomas	*COX-2↑*	(70)
	Epithelial cells	Breast cancer	*Twist↑*	(78)
		Breast cancer	*Snail↑*	(78)
		Breast cancer	*Slug↑*	(78)
		Breast cancer	*Sip1↑*	(78)
	Endothelial cells	Breast cancer	*MMP-9↑*	(86)
		Lung carcinoma	*VEGF↑*	(89)
		breast cancer, colorectal cancer, pancreatic cancer	CXCL12*↑*	(91, 92)
		Anaplastic thyroid carcinoma	*MCP1↑*	(94, 107)
Angiogenesis and Invasion		Pancreatic tumor, Prostate tumor, Breast cancer	*MMP-9↑*	(99, 100, 108)
		Prostate tumor, Ovarian cancer	*VEGF↑*	(58, 79, 80)
		Melanoma	*CXCL12↑*	(91–93)
		Anaplastic thyroid carcinoma	*MCP1/CCL2↑*	(107, 109)
		Anaplastic thyroid carcinoma	*IL-8/CXCL8↑*	(107, 109, 110)
		Pancreatic tumor, Breast cancer	*uPA(pro-uPA)↑*	(103–105)
Cancer related inflammation		Colitis-associated cancer	*IL-6↑*	(111, 112)
		Pancreatic tumor	*IL-1β↑*	(113)
Crosstalk	STAT3	Colon cancer	*S100A9↑*	(66, 114)
		Colitis-associated cancer	*IL-6↑*	(93)
		Colitis-associated cancer	*COX-2↑*	(5, 33, 70, 115)

TAMs, Tumor-Associated Macrophages; DCs, Dendritic cells; CAFs, Cancer-associated fibroblasts; IL-1,6, Interleukin-1,6; LT, lymphotoxin; NOS, Nitric Oxide Synthase; MMP-9, Matrix metalloproteinase-9; COX-2, Cyclooxygenase-2; MCP-1, monocyte chemotactic protein-1; TNF-α, Tumor Necrosis Factor Alpha; HIF-α, Hypoxia-inducible factor-alpha; CXCL8, C-X-C motif chemokine ligand-8; CCL20, Chemokine Ligand 20; uPA, Urokinase‐type plasminogen activator.

## 5 Crosstalks between different cells regulated by NF-κB in TME

Just as we concluded and reviewed above, TME is a highly complicated system mainly composed of infiltrating immune cells, cancer-associated stromal cells(e.g. CAFs, ECs) and tumor cells, along with the extracellular matrix (ECM) and various molecules (116). It is important to note that their reciprocal interactions either amplify or counteract their effects, giving rise to a complex network within the tumor microenvironment which ultimately governs the occurrence and development of tumors.

The significance of effector T cells as crucial anti-tumor agents within the tumor microenvironment is widely acknowledged, despite their frequent manifestation of anergy or exhaustion. Nevertheless, the other cells in TME present in the tumor microenvironment can modulate effector T cells capacity through interaction and crosstalks, thereby offering potential avenues for ameliorating T cell exhaustion. The dendritic cells (DCs) in tumor-draining lymph nodes of cancer patients can disrupt immunological tolerance by presenting antigens to naive T cells. We now understand that interactions between T cells and DCs not only play a critical role in the cancer immunity cycle, including important reactions within the tumor microenvironment that support anti-tumor responses, but also are essential for controlling tumor and successful cancer immunotherapy (54). So due to the critical role of DCs in shaping the activity of CD8^+^T cells, increasing effort has been made to repair and enhance the insufficient T cells. The CCR7^+^DCs that metastasize through lymph nodes migrate to lymph node regions abundant in CD8^+^ and CD4^+^ T cells by binding to CLL19 and CCL21 on the cortical surface of lymph nodes(LN). Upon binding antigen peptide-MHC via TCR-CD28 and costimulatory receptors, T cells trigger an immune response, leading to sustained and stable interaction with cDCs in the lymph node region. This promotes cytokine communication between them, resulting in positive feedback that further amplifies the effector T cell-mediated immune effect (54). We have mentioned in the section 3.5, in melanoma model, NF-κB regulate the transcription factor IRF1,which is essential in the development of CCR7^+^DCs from cDC1s (61). This indicates NF-κB functions as anti-tumoral role through accelerating DCs-T cells interaction. Similarly, Christopher S. Garris found that DCs in TME can sense IFN-γ,which is produce by aPD-1 activated T cells. In turn, DCs produce more IL-12 to enhance the anti-tumor effect of T cells, which is proven to be regulated by non-canonical NF-κB transcription factor pathway (106). Through generating transgenic mice with targeted NIK deletion in CD11c^+^ DCs, Anand K. Katakam found that non-canonical NF-κB mediated by NIK is indispensable for DCs to cross-present antigen and initiate CD8^+^T cells responses to CD40 agonism (117).

Other interactions with different cell types happen in TME as well. a study demonstrated that CAFs is involved in the abnormal differentiation and impaired antigen presenting function of DCs via down-regulating of NF-κB (118). In this aspect, NF-κB appears to play a beneficial role in the anti-tumoral activity by facilitating intercellular communication among different cell types within the tumor microenvironment (TME). Therefore, further investigation is needed to determine whether NF-κB also regulates other crucial immune cell interactions such as B cells-T cells.

## 6 Linking chronic inflammation to the progression of cancer

Chronic inflammation is widely recognized as a hallmark of cancer, driving tumor progression. NF-κB, a critical inflammatory signaling pathway, acts as a tumor promoter in many cancer types. The inflammatory microenvironment supports tumor growth by enhancing cellular proliferation, survival, migration, and angiogenesis (119). Pro-inflammatory cytokines like TNF-α and IL-6 play key roles in promoting tumor progression.

Notably, in the Mdr2-/- knockout mice model, a model of CAC, a seminal study demonstrated that inhibiting NF-κB signaling in inflammatory and endothelial cells—through IkB-superrepressor induction or anti-TNF-α treatment reverses the process of transformed hepatocytes developing to hepatocellular carcinoma (120), which represented the pioneering attempt to investigate the involvement of NF-κB in both inflammatory processes and carcinogenesis.IL-6, another key factor in this process, is released by myeloid cells under the control of NF-κB and influences various aspects of tumor proliferation (121). Studies have shown that NF-κB signaling can enhance tumor growth both directly and indirectly. Inhibiting IKK-β, a crucial component of the NF-κB pathway, reduces the production of inflammatory mediators like IL-6 and TNF-α, thereby limiting inflammation-driven cell proliferation in CAC (111). Further studies have revealed that the tumor pro-proliferative effects of NF-κB are mediated indirectly through IL-6 and related cytokines produced by myeloid cells. These cytokines activate STAT3 in IECs, affecting both their survival and proliferation (112). Additionally, research has highlighted the role of nitric oxide (NO) in fostering chemoresistance in pancreatic cancer by stimulating IL-1β secretion in tumor cells, thereby safeguarding them against anticancer drugs. This mechanism involves a paracrine-positive feedback loop that activates NF-κB, underlining the complex interplay between inflammation and cancer progression (113).

However, we must admit that the inflammation process is constantly evolving and the effect of NF-κB on cancer is not always positive. So it is complicated and tricky to understand the specific impact of NF-κB on the process of cancer. While NF-κB is a central regulator of gene expression in both innate and adaptive immunity, contributing to the elimination of transformed cells, its function varies contextually. In acute inflammation, NF-κB activation within immune cells often exhibits potent cytotoxic effects against tumor cells (122). Earlier research also reported that in human squamous cell carcinomas (SCCs), co-expression of IκBα, an inhibitor of NF-κB, fires the process of Ras-induced cancer (123), which is consistent with the susceptibility of blockade of NF-κB to squamous cell carcinoma (124). Contradictions emerge regarding the role of IKKβ across different cancer types. In a melanoma mouse model, the deletion of IKKβ in myeloid cells leads to enhanced tumor growth due to the change of myeloid cytokine/chemokine expression (125). Conversely, in a CAC model, the loss of IKKβ in enterocytes accompanies with a diminishment of tumor size even in the presence of heightened inflammation (126). The dual effect of IKKβ may partly account for the lack of clinical success of IKKβ inhibitors to date.

In a chemically induced liver cancer model, the deletion of NEMO in hepatocytes led to spontaneous hepatocellular carcinoma development in mice, suggesting NEMO functions as a tumor suppressor in the liver, revealing a function of NEMO as a tumor suppressor in the liver. However, this effect still requires the activation of NF-κB in Kupffer cells, which induces the expression of cytokines and chemokines (127). This may be explained by the anti-apoptosis character of NF-κB. The deficiency of NEMO in hepatocytes undergoes apoptosis in response to the chemical stimulation which triggers the compensatory proliferation of Kupffer cells and creates a tumor microenvironment associated with inflammation to promote tumor development. The tricky phenomenon may reflect that different cancer systems or different progress of cancer correspond to the involvement of different polarized inflammatory reactions induced by NF-κB. Considering the various effects of NF-κB on tumor progression, it must be cautious to target NF-κB numbers and tip the balance in different biological activity in order to treat cancer.

## 7 Crosstalks with STAT3 and Wnt/β-catenin signaling pathway

STAT3 plays a pivotal role in various tumor-related processes, including cell proliferation, survival, angiogenesis, and invasion. It is also a crucial factor in tumor-induced immunosuppression at multiple levels (128). In many types of tumor,NF-κB and STAT3 are constitutively activated in response to the upstream autocrine and paracrine factors that are produced within the tumor microenvironment.When receiving the stimulis such as cytokines and growth factors, NF-κB and STAT3 influence the progression of tumor through regulating repression of cytokines, growth factors in tumor cells and some other inflammatory/immune mediators (115).

The most important fact of NF-κB and STAT3 is to regulate the expression of cytokines chemokines and chemoattractants which act in the recruitment and renewal of different cells in the tumor microenvironment. First of all, STAT3-inducible up-regulation of the myeloid-related protein S100A9 enhances MDSC accumulation, which leads to suppression of anti-tumor immune responses (66). Another research proved that S100A8/A9-enhanced NADPH oxidase affects downstream NF-κB signaling pathway, which may play role in positive effect on MDSCs recruitment (114).

NF-κB and STAT3 act as two major transcriptional factors to link inflammation with tumorigenesis, and they functionally interact with each other at many different layers (33). The interaction between NF-κB and STAT3, however, is complex, as they act as cooperative partners in regulating a variety of target genes that influence tumor progression (33). Key downstream factors of NF-κB such as IL-6 and cyclooxygenase-2 (COX2) not only play critical roles in tumor initiation and progression under the influence of NF-κB but are also involved in STAT3 activation (128). Intriguingly, STAT3 has been shown to mediate the acetylation of NF-κB, enhancing its nuclear retention through the recruitment of acetyltransferase p300.STAT3-mediate acetylation activity leads to continuous NF-κB, which is crucial for alter of tumor microenvironment. On the another hand, constitutive activation of NF-κB results in more secretion of IL-6,which also activates STAT3 and formats a positive activation loop. Moreover, phosphorylated STAT3 has been reported to activate the transcription of proliferative genes through NF-κB, suggesting a positive feedback loop within the NF-κB-IL-6-STAT3 signaling pathway (69). This pathway, as discussed, influences the expression of pro-survival and angiogenic factors like VEGF and MMP9, which are also regulated by STAT3 (79, 128, 129). Additionally, both NF-κB and STAT3 act as transcriptional repressors of p53 expression, a critical tumor suppressor factor in the tumor microenvironment (TME) (69, 130).

Although active NF-κB in immune cells and tumor cells have a crucial role in anti-tumor activity, constitutive activation of NF-κB in tumor cell is recognized as a promoter of pro-survival and angiogenic factors, such as BCL-XL,surviving,MCL1,VEGF and MMP9,which are also regulated by STAT3 (5). It is also proved that STAT3 inhibit IL-12, TNF, IFNβ, CXCL10, CCL5 (also known as RANTES), CD40, CD80, CD86 and MHC class II molecules, which are immune-stimulating genes regulated by NF-κB (131, 132). In this aspect, the relationship of NF-κB and STAT3 seem to be confounding. Another interesting research proved that absence of IKKβ causes STAT3 activation, leading to upregulated ROS accumulation in mouse hepatocellular carcinomas (HCC), and inverse relationships between the activation of NF-κB and STAT3 have also been observed in human HCC (133).

Another important crosstalk of NF-κB is Wnt/β-catenin signaling pathway, which regulates stem cell renewal, organogenesis, cell cycle and inflammation environment of tumor (134, 135). It is now accepted that the abnormal of Wnt/β-catenin signaling pathway is a early event of colorectal tumorigenesis (136, 137). Sarah Schwitalla et al.found NF-κB(p65) directly binds to β-catenin, which increase the expression of Wnt-regulated stem cell gene in IEC, leading to the dedifferentiation of epithelial nonstem cells into tumor-initiating cells (75).

## 8 The connections of NF-κB and metabolism within TME

### 8.1 Glycolysis

Metabolic reprogramming is a hallmark of tumor cells, allowing them to meet the material and energy demands of rapid tumor cells proliferation. According to the As outlined by the Warburg hypothesis, cancer cells heavily depend on glycolysis as their main energy source. TME favors anaerobic glycolysis, leading researchers to investigate it as a potential therapeutic target (138, 139). A compelling aspect of NF-κB signaling is its ability to reprogram cellular metabolic networks, thereby sustaining tumor proliferation and driving cancer progression.

Evidence suggests that NF-κB regulates glycolysis in the TME. Rel-A, a key NF-κB subunit, also plays a role in glycolysis regulation. The lack of Rel-A in mouse embryonic fibroblasts (MEFs) shows increasement of glucose consumption and lactate production, which indicates that NF-κB is involved in the process of reprogramming to glycolysis. Furthermore, Rel-A deficient impair the ability of adaptation to glucose starvation and lead to the death of cell instead of reprogramming to OXPHOS (139). Kawauchi et al. found that IKK/NF-κB pathway activation caused increased expression of GLUT3, enhancing glucose uptake and promoting glycolysis. Interestingly, a positive feedback loop between glycolysis and the IKK/NF-κB pathway supports oncogenic transformation driven by H-Ras (140). Interestingly, IKKβ functions as a sensor of glutamine levels in the TME, supporting cancer cell survival through various mechanisms. When glutamine levels are low, IKKβ is activated independently of NF-κB. Through combining with key components of glycosis,such as 6-phosphofructo-2-kinase/fructose-2,6biphosphatase isoform 3 (PFKFB3) and glutamate dehydrogenase 1 (GDH1),IKKβ plays a critical role in inhibiting glycolysis under low glutamine (141).

NF-κB is also a mediator hub of shaping T cell response via reprogramming the metabolism of TME.A study identified that NF-κB-inducing kinase (NIK) stabilize hexokinase 2 (HK2), a rate-limiting enzyme of the glycolytic pathway, by regulating the ROS level and eventually balance the NADPH redox system. The specific deletion of HK2/NIK in mice display impaired aerobic glycolysis and a dysregulated response of T cell to acute infection (25).

### 8.2 Lipid metabolism

Lipid is an important part of biological membranes and constitution of cells and it is also used by an energy storage and metabolism. Furthermore, lipids also regulate various cellular processes such as uptake, synthesis, and hydrolysis. In the TME, lipid metabolism is reprogrammed to meet the demands of tumor cells, supporting their rapid proliferation, survival, migration, invasion, and metastasis (142).

In gastric cancer, it was proved that FA-induced hyper-O-GlcNAcylation promotes the expression of CD36 by increasing the activity of NF-κB and directly modifying CD36 at S468 and T470,which is convenient for the metastasis of gastric cancer (143). The deficiency of Rel-A in MEFs and human CRC cell lines leads to the alteration of lipidomic profiles via carboxylesterase 1(CES1), connecting obesity-related inflammation with lipid metabolism in aggressive forms of CRC (144). In *Drosophil*, NF-κB/Relish and Foxo competitively regulate the balance lipid metabolism during metabolic adaptation. Although the process is independent of infection, Relish-Foxo signaling still mediates triglyceride catabolism under chronic bacterial infection, which may also contribution to tumor-associated inflammation (145).

As an important member of NF-κB family, c-Rel is considered as a hazard of cancer and inflammation, which is proved by the fact of lacking c-Rel reduces susceptibility to infectious diseases. A genome-wide associated studies shows that, comparing to control, Rel^−/−^ MDSCs show difference expression of various genes involved in glucose, amino acid, and lipid metabolism and cell cycle checkpoint and proinflammatory activity (64).

GADD45β is a member of the “growth arrest and DNA damage-inducible” (GADD45) gene family. It was identified as a regulator of live fatty acid under fasting stress and keep the balance of normal metabolism of chronic nutrient oversupply (146). In one hand,GADD45β is proved to regulate NF-κB to play a role in antiapoptotic activity in cancer cells. On the other hand, the deletion of GADD45β in MDSCs restores the activation of TAMs and CD8^+^ T cells infiltration and ultimately hinders the process of tumorgenesis (147).

### 8.3 Oxidative metabolism and mitochondrial metabolism

In normal cells, glucose deprivation triggers the activation of AMP-activated protein kinase (AMPK), an energy sensor that reprograms cellular metabolism toward fatty acid oxidation and OXPHOS to meet the bioenergetic needs of the cell and maximize energy efficiency (148).

In energy-deficient environments, AMPK, an energy sensor, shifts cellular metabolism towards fatty acid oxidation and OXPHOS to optimize energy production. OXPHOS, a key component of mitochondrial metabolism, is often dysregulated and reprogrammed in malignant tumor cells. This metabolic plasticity is exploited by cancer cells, contributing to tumorigenesis (149).

Just as we demonstrated in section 3.2, regulation of NF-κB for metabolic reprogramming OXPHOS and glycolysis in MDSC, which alongside with the change of checkpoint such as PD-1 in MDSC, which involves in anti-immunity such as CD8^+^T cells responses. Ting Li et al. found that comparing to wild type cells, Rel^−/−^ MDSCs displayed diminishing OXPHOS flux and mitochondrial ATP production, while increasing glycolysis. While C/EBPβ overexpression in Rel knockout MDSCs isolated from LysM-Cre/RelF/F mice effectively rescued their phenotype, reducing glycolysis and increasing OXPHOS and expression of proinflammatory cytokines (28). These results align with the concept that OXPHOS-based metabolism is a hallmark of immunosuppressive cells, such as Tregs and M2 TAMs (150). Consistently, the inhibition of NF-κB/Rel-A in MEFs resulted in decreased oxygen consumption and glycolytic reprogramming, with augmented glucose consumption and lactate production, which is reversed by p53 reconstitution in Rel-A^−/−^ cells, suggesting the indispensable role of NF-κB/p53 axis in metabolic adaptation in normal cells and cancer (149).

## 9 Role of NF-κB in the resistance to therapy via TME

It is well documented that NF-κB signaling pathway functions in enhancing drug resistance in chemotherapy, immunotherapy, endocrine, and targeted therapy. Increasing numbers of studies proved that chemotherapy for cancer also depends on the interaction between cancer cells and the surrounding TME components (151). We will conclude the mechanism of resistance induced by NF-κB in different cell components of TME.

Just we mentioned above, TAMs as the most abundant population of tumor-infiltrating immune cells within TME, are generally divided into two subsets– pro-inflammatory classical (M1) and suppressive alternatively activated (M2) subtypes. In most cases, just as we mentioned above, the activation of NF-κB induces the M2 macrophage in TME, which is a promoter of tumor progression (152). TNFα secreting from TAMs promotes melanoma resistance to MAPK pathway inhibitors through NF-κB via regulation of expression of the microphthalmia transcription factor (MITF) (153). Another report found that in the duration of antitumor immunity by inducing interferon (IFN) response, the activation of NF-κB by a long noncoding RNA-IFN-responsive nuclear factor-κB activator (IRENA) in M1 macrophages and increased secretion of pro-inflammatory cytokines, which promote breast cancer chemoresistance (154). In human ovarian cancer (OC) cells, NF-κB mediates the enhanced expression and production of CCL2 and stimulates the activation of the PI3K/Akt pathway, which results in the development of paclitaxel resistance. During this process, CCL2 also functions as a chemotactic factor, inducing macrophage chemotaxis, which may lead to chemotherapy resistance (155). Another research proved triptolide(TPL) could inhibit the migration and invasion of OC cells *in vitro* and *in vivo* by inhibiting the polarization of M2 TAMs, which reduced the tumor burden via PI3K/Akt/NF-κB signaling pathway (156). Using 10x Genomics single-cell sequencing technology, CCL5 was increased secreted undergoing aPKCι-induced EMT and consequently modulated macrophage recruitment and polarization dependent on NF-κB signaling, which eventually leads to gemcitabine resistance (157).

As a major component of the tumor stroma, CAFs function in the dialogue between tumor cells and the TME, playing a central role in tumor progression, including tumorigenesis, supporting angiogenesis, fostering resistance to therapy, and suppressing antitumor immune responses. circZFR was highly expressed in cisplatin (DDP)-resistant HCC cell lines and the CAFs-derived exosome, regulating DDP resistance of the HCC cells via STAT3/NF-κB signaling (158). Lnc RNA UPK1A-AS1 induced by IL8/NF-κB signaling in CAFs serves as a chemoresistance promotor and is critical for active IL8-induced oxaliplatin resistance in pancreatic ductal adenocarcinoma (PDAC) (159). Similarly, CAF-derived IL-8 promotes chemoresistance in human gastric cancer via NF-κB activation (160).

MDSCs activated the PI3K/AKT NF-κB signaling pathway in B cells through the PD-1/PD-L1 axis, which forms the immunosuppressive functions of PD-1^+^PD-L1^+^ B cells, which suggests the role of NF-κB in PD-1 therapy resistance and the combined targeting of PI3K/AKT and PD-1/PD-L1 tactics (161). In T cells, the deletion of CARMA1 or inhibition of MALT1 enhances the sensitivity to the PD-1 blockade therapy via inducing IFN-γ-secretion in TME, which we just mentioned in section 3.2 (162).

Considering the positive effect of NF-κB in different components of TME to therapy resistance, the research found strategies for reversing drug resistance mainly involved in NF-κB inhibitors, which showed promising outcomes in preclinical experiments although there is a long distance to get the clinical application before finding a better balance between positive and negative effect.

## 10 Discussion

A wealth of research in recent years has been paid attention to the role of NF-κB in the orchestration and dynamics of TME. In this review, we intend to highlight the pleiotropic role of the NF-κB signaling pathway in more aspects of TME. NF-κB signaling pathway appears to be crucial in shaping tumor immune microenvironment, changing the function of stromal cells of TME, regulating angiogenesis and invasion and linking inflammation with tumorgenesis, leading to directly or indirectly influence the dynamics switch between anti-tumoral activity and pro-tumoral activity. Although several fundamental research findings presented in this review demonstrate the crucial role of NF-κB signaling in orchestrating the tumor microenvironment, it is important to acknowledge that potential biases may exist due to the methodology employed in these studies, thereby causing confusion regarding the precise involvement of NF-κB in the tumor microenvironment. Firstly, part of the NF-κB signaling involved in the shaping of cytokines affecting the tumor microenvironment has only been verified in tumor cell lines (109). Additionally, RNA sequencing has been employed in several studies to investigate disparities in gene expression among cancer patients (57), with a specific focus on enhancing the NF-κB signaling pathway. However, it is imperative to further explore and elaborate upon these findings due to the potential overlap between signaling pathways and the distinct roles played by certain genes.

A comprehensive understanding of the complicated role of NF-κB signaling in TME entitles researchers to better explain the effect of therapies targeting NF-κB signaling. It is well documented that TNF-αinducing NF-κB signaling and regulate the expression of anti-apoptosis and cell cycle progression genes. So inhibition of NF-κB or anti-TNF-α may offer an attractive combined strategy for immunomodulatory cancer therapy. p50 knockout macrophages exhibited more T cell infiltration and higher expression of pro-inflammatory cytokines *in vivo*, which enlightens us that targeting p50 represents an innovative anticancer strategy, complementary to immunostimulatory strategies (46). In melanoma and lymphoma,c-Rel is proven to play a central role in aTreg and MDSC biology and suggests that c-Rel deletion has effects on anti-tumor responses (64). Pentoxifylline (PTXF), an FDA-approved drug, can cause selective degradation of c-Rel, without affecting p65.PTXF has been widely used in the therapy of type 2 diabetes mellitus and chronic kidney disease (CKD). Although the role of c-Rel in shaping of Treg and MDSC in TME is well acknowledged, the inhibitor of c-Rel such as PTXF or R96A has not been used in the clinical trial. What is more, given the importance of CARMA1-BCL10-MALT1 as an NF-κB platform complex in lymphocyte development and function, multiple studies have confirmed that MALT1 suppression in Treg cells will reshape the immune microenvironment convenience to immune checkpoint therapy (162). Of note, IKKβ inhibitors have demonstrated efficacy in various pre-clinical models of cancer and inflammatory disease. For instance, MLN-120B(the ATP-competitive IKKβ inhibitor) is convinced to be a promoter of therapeutic efficiency in mouse models of rheumatoid arthritis(RA). Furthermore, clinical trials of the drugs inhibiting IKKβ are still rare in considering the various reasons, including the important role of IKKβ in some cases, improper dose plan and impertinent patient group selection (163). In contrast to the fact of tumor-promoting functions of NF-κB in both malignant and inflammatory immune cells, the activation of the IKKβ/NF-κB signaling axis in CAFs was surprisingly found to be a tumor suppressor of intestinal tumor growth (72).

The complexity and heterogeneity of the TME also pose substantial challenges to such targeted approaches. For instance, TAMs exhibit varied phenotypes based on their differentiation, significantly impacting tumor evolution and the TME (164, 165). Moreover, the specificity of the TME to each tumor’s organ or tissue of origin, coupled with the pre-existing conditions such as chronic inflammation in cancers of the colon, stomach, and liver (as opposed to gliomas and breast cancers), further complicates therapeutic interventions (166). It is also crucial to acknowledge the sophisticated feedback mechanisms regulating NF-κB activation, necessitating a cautious and well-considered approach in leveraging NF-κB targeting as a cancer treatment strategy.

The fundamental principles and findings from prior clinical trials collectively indicate that NF-κB is more likely to have side effects. This consensus is based on the fact that NF-κB plays an indispensable role in many biological processes, including immune cell development, normal cell proliferation, and so on, and its inhibition may produce undesirable side effects. So before we can safely put targeting or inhibiting NF-κB to clinical application, we must realize the putative efficacy mainly depends on the type of cancer and the dynamics immune microenvironment. But during the dynamic changing of NF-κB in TME, the short-term inhibitor of this signaling in the peak range may be efficient in the therapy of cancer. For example, as a characteristic of activated B cell-like Diffuse large B-cell lymphoma (ABC DLBCL), constitutive activation of NF-κB signaling drives cancer cell proliferation/survival (167), which provides a shred of reasonable evidence for therapeutic strategies targeting IKKβ/NF-κB. On the other side, it is still not an advisable therapy to singly inhibit NF-κB signaling number to achieve the clinical expectation. The use of synergistic combinations between targeting NF-κB and other immune therapy is to expected be employed to achieve a desired therapeutic effect, thus reducing systemic toxicity. Just as we mentioned above, in some cases, NF-κB may serve as a tumor suppressor, which suggests it must be cautious to control the duration and dosage of this treatment plan.

## References

[1] Vitale I Manic G Coussens LM Kroemer G Galluzzi L. Macrophages and metabolism in the tumor microenvironment. Cell Metab. (2019) 30:36–50. doi: 10.1016/j.cmet.2019.06.001 31269428

[2] Marozzi M Parnigoni A Negri A Viola M Vigetti D Passi A. Inflammation, extracellular matrix remodeling, and proteostasis in tumor microenvironment. Int J Mol Sci. (2021) 22:8102. doi: 10.3390/ijms22158102 34360868 PMC8346982

[3] Naser R Fakhoury I El-Fouani A Abi-Habib R El-Sibai M. Role of the tumor microenvironment in cancer hallmarks and targeted therapy (Review). Int J Oncol. (2023) 62:23. doi: 10.3892/ijo.2022.5471 36579669

[4] Wan F Lenardo MJ. The nuclear signaling of NF-kappaB: current knowledge, new insights, and future perspectives. Cell Res. (2010) 20:24–33. doi: 10.1038/cr.2009.137 19997086 PMC3052775

[5] Karin M Greten FR. NF-kappaB: linking inflammation and immunity to cancer development and progression. Nat Rev Immunol. (2005) 5:749–59. doi: 10.1038/nri1703 16175180

[6] Taniguchi K Karin M. NF-κB, inflammation, immunity and cancer: coming of age. Nat Rev Immunol. (2018) 18:309–24. doi: 10.1038/nri.2017.142 29379212

[7] Sun SC Ley SC. New insights into NF-kappaB regulation and function. Trends Immunol. (2008) 29:469–78. doi: 10.1016/j.it.2008.07.003 PMC5751948 18775672

[8] Steinberg GR Hardie DG. New insights into activation and function of the AMPK. Nat Rev Mol Cell Biol. (2022). doi: 10.1038/s41580-022-00547-x 36316383

[9] Vallabhapurapu S Karin M. Regulation and function of NF-kappaB transcription factors in the immune system. Annu Rev Immunol. (2009) 27:693–733. doi: 10.1146/annurev.immunol.021908.132641 19302050

[10] Hayden MS Ghosh S. Shared principles in NF-kappaB signaling. Cell. (2008) 132:344–62. doi: 10.1016/j.cell.2008.01.020 18267068

[11] Yu H Lin L Zhang Z Zhang H Hu H. Targeting NF-κB pathway for the therapy of diseases: mechanism and clinical study. Signal Transduction Targeting Ther. (2020) 5:209. doi: 10.1038/s41392-020-00312-6 PMC7506548 32958760

[12] Gaptulbarova KA Tsyganov MM Pevzner AM Ibragimova MK Litviakov NV. NF-kB as a potential prognostic marker and a candidate for targeted therapy of cancer. Exp Oncol. (2020) 42:263–9. doi: 10.32471/10.32471/exp-oncology.2312-8852.vol-42-no-4 33355866

[13] Fantini M Arlen PM Tsang KY. Potentiation of natural killer cells to overcome cancer resistance to NK cell-based therapy and to enhance antibody-based immunotherapy. Front Immunol. (2023) 14:1275904. doi: 10.3389/fimmu.2023.1275904 38077389 PMC10704476

[14] Rousalova I Krepela E. Granzyme B-induced apoptosis in cancer cells and its regulation (review). Int J Oncol. (2010) 37:1361–78. doi: 10.3892/ijo_00000788 21042704

[15] Zhou J Zhang J Lichtenheld MG Meadows GG. A role for NF-kappa B activation in perforin expression of NK cells upon IL-2 receptor signaling. J Immunol Baltim. Md 1950. (2002) 169:1319–25. doi: 10.4049/jimmunol.169.3.1319 12133954

[16] Huang C Bi E Hu Y Deng W Tian Z Dong C. A novel NF-kappaB binding site controls human granzyme B gene transcription. J Immunol Baltim. Md 1950. (2006) 176:4173–81. doi: 10.4049/jimmunol.176.7.4173 16547254

[17] Bernareggi D Xie Q Prager BC Yun J Cruz LS Pham TV. CHMP2A regulates tumor sensitivity to natural killer cell-mediated cytotoxicity. Nat Commun. (2022) 13:1899. doi: 10.1038/s41467-022-29469-0 35393416 PMC8990014

[18] Fusella F Seclì L Brancaccio M. Escaping NK cells and recruiting neutrophils: How Morgana/NF-κB signaling promotes metastasis. Mol Cell Oncol. (2018) 5:e1432258. doi: 10.1080/23723556.2018.1432258 30250889 PMC6150045

[19] Fusella F Seclì L Busso E Krepelova A Moiso E Rocca S. The IKK/NF-κB signaling pathway requires Morgana to drive breast cancer metastasis. Nat Commun. (2017) 8:1636. doi: 10.1038/s41467-017-01829-1 29158506 PMC5696377

[20] Gerondakis S Fulford TS Messina NL Grumont RJ. NF-κB control of T cell development. Nat Immunol. (2014) 15:15–25. doi: 10.1038/ni.2785 24352326

[21] Paul MS Ohashi PS. The roles of CD8+ T cell subsets in antitumor immunity. Trends Cell Biol. (2020) 30:695–704. doi: 10.1016/j.tcb.2020.06.003 32624246

[22] Evaristo C Spranger S Barnes SE Miller ML Molinero LL Locke FL. Cutting edge: engineering active IKKβ in T cells drives tumor rejection. J Immunol Baltim. Md 1950. (2016) 196:2933–8. doi: 10.4049/jimmunol.1501144 PMC4799771 26903482

[23] Giordano M Roncagalli R Bourdely P Chasson L Buferne M Yamasaki S. The tumor necrosis factor alpha-induced protein 3 (TNFAIP3, A20) imposes a brake on antitumor activity of CD8 T cells. Proc Natl Acad Sci U S A. (2014) 111:11115–20. doi: 10.1073/pnas.1406259111 PMC4121810 25024217

[24] Hopewell EL Zhao W Fulp WJ Bronk CC Lopez AS Massengill M. Lung tumor NF-κB signaling promotes T cell-mediated immune surveillance. J Clin Invest. (2013) 123:2509–22. doi: 10.1172/JCI67250 PMC3668836 23635779

[25] Gu M Zhou X Sohn JH Zhu L Jie Z Yang J-Y. NF-κB-inducing kinase maintains T cell metabolic fitness in antitumor immunity. Nat Immunol. (2021) 22:193–204. doi: 10.1038/s41590-020-00829-6 33398181 PMC7855506

[26] Speiser DE Chijioke O Schaeuble K Münz C. CD4+ T cells in cancer. Nat Cancer. (2023) 4:317–29. doi: 10.1038/s43018-023-00521-2 36894637

[27] Lalle G Lautraite R Bouherrou K Plaschka M Pignata A Voisin A. NF-κB subunits RelA and c-Rel selectively control CD4^+^ T cell function in multiple sclerosis and cancer. J Exp Med. (2024) 221. doi: 10.1084/jem.20231348 PMC10986815 38563819

[28] Oh H Grinberg-Bleyer Y Liao W Maloney D Wang P Wu Z. An NF-κB transcription-factor-dependent lineage-specific transcriptional program promotes regulatory T cell identity and function. Immunity. (2017) 47:450–465.e5. doi: 10.1016/j.immuni.2017.08.010 28889947 PMC5679261

[29] Grinberg-Bleyer Y Oh H Desrichard A Bhatt DM Caron R Chan TA. NF-κB c-rel is crucial for the regulatory T cell immune checkpoint in cancer. Cell. (2017) 170:1096–1108.e13. doi: 10.1016/j.cell.2017.08.004 28886380 PMC5633372

[30] Ruland J Hartjes L. CARD-BCL-10-MALT1 signalling in protective and pathological immunity. Nat Rev Immunol. (2019) 19:118–34. doi: 10.1038/s41577-018-0087-2 30467369

[31] Qi T Luo Y Cui W Zhou Y Ma X Wang D. Crosstalk between the CBM complex/NF-κB and MAPK/P27 signaling pathways of regulatory T cells contributes to the tumor microenvironment. Front Cell Dev Biol. (2022) 10:911811. doi: 10.3389/fcell.2022.911811 35927985 PMC9343696

[32] Hashimoto M Kamphorst AO Im SJ Kissick HT Pillai RN Ramalingam SS. CD8 T Cell exhaustion in chronic infection and cancer: opportunities for interventions. Annu Rev Med. (2018) 69:301–18. doi: 10.1146/annurev-med-012017-043208 29414259

[33] Fan Y Mao R Yang J. NF-κB and STAT3 signaling pathways collaboratively link inflammation to cancer. Protein Cell. (2013) 4:176–85. doi: 10.1007/s13238-013-2084-3 PMC4875500 23483479

[34] Kamphorst AO Wieland A Nasti T Yang S Zhang R Barber DL. Rescue of exhausted CD8 T cells by PD-1-targeted therapies is CD28-dependent. Science. (2017) 355:1423–7. doi: 10.1126/science.aaf0683 PMC5595217 28280249

[35] Capece D Verzella D Flati I Arboretto P Cornice J Franzoso G. NF-κB: blending metabolism, immunity, and inflammation. Trends Immunol. (2022) 43:757–75. doi: 10.1016/j.it.2022.07.004 35965153

[36] Pohl T Gugasyan R Grumont RJ Strasser A Metcalf D Tarlinton D. The combined absence of NF-kappa B1 and c-Rel reveals that overlapping roles for these transcription factors in the B cell lineage are restricted to the activation and function of mature cells. Proc Natl Acad Sci U S A. (2002) 99:4514–9. doi: 10.1073/pnas.072071599 PMC123679 11930006

[37] Downs-Canner SM Meier J Vincent BG Serody JS. B cell function in the tumor microenvironment. Annu Rev Immunol. (2022) 40:169–93. doi: 10.1146/annurev-immunol-101220-015603 35044794

[38] Ammirante M Kuraishy AI Shalapour S Strasner A Ramirez-Sanchez C Zhang W. An IKKα-E2F1-BMI1 cascade activated by infiltrating B cells controls prostate regeneration and tumor recurrence. Genes Dev. (2013) 27:1435–40. doi: 10.1101/gad.220202.113 PMC3713424 23796898

[39] Wu K Lin K Li X Yuan X Xu P Ni P. Redefining tumor-associated macrophage subpopulations and functions in the tumor microenvironment. Front Immunol. (2020) 11:1731. doi: 10.3389/fimmu.2020.01731 32849616 PMC7417513

[40] Forssell J Oberg A Henriksson ML Stenling R Jung A Palmqvist R. High macrophage infiltration along the tumor front correlates with improved survival in colon cancer. Clin Cancer Res. (2007) 13:1472–9. doi: 10.1158/1078-0432.CCR-06-2073 17332291

[41] Gordon S Martinez FO. Alternative activation of macrophages: mechanism and functions. Immunity. (2010) 32:593–604. doi: 10.1016/j.immuni.2010.05.007 20510870

[42] Condeelis J Pollard JW. Macrophages: obligate partners for tumor cell migration, invasion, and metastasis. Cell. (2006) 124. doi: 10.1016/j.cell.2006.01.007 16439202

[43] Ong S-M Tan Y-C Beretta O Jiang D Yeap W-H Tai JJY. Macrophages in human colorectal cancer are pro-inflammatory and prime T cells towards an anti-tumour type-1 inflammatory response. Eur J Immunol. (2012) 42:89–100. doi: 10.1002/eji.201141825 22009685

[44] Hagemann T Lawrence T McNeish I Charles KA Kulbe H Thompson RG. “Re-educating” tumor-associated macrophages by targeting NF-kappaB. J Exp Med. (2008) 205:1261–8. doi: 10.1084/jem.20080108 PMC2413024 18490490

[45] Ryan AE Colleran A O’Gorman A O’Flynn L Pindjacova J Lohan P. Targeting colon cancer cell NF-κB promotes an anti-tumour M1-like macrophage phenotype and inhibits peritoneal metastasis. Oncogene. (2015) 34:1563–74. doi: 10.1038/onc.2014.86 24704833

[46] Saccani A Schioppa T Porta C Biswas SK Nebuloni M Vago L. p50 nuclear factor-kappaB overexpression in tumor-associated macrophages inhibits M1 inflammatory responses and antitumor resistance. Cancer Res. (2006) 66:11432–40. doi: 10.1158/0008-5472.CAN-06-1867 17145890

[47] Kiriakidis S Andreakos E Monaco C Foxwell B Feldmann M Paleolog E. VEGF expression in human macrophages is NF-kappaB-dependent: studies using adenoviruses expressing the endogenous NF-kappaB inhibitor IkappaBalpha and a kinase-defective form of the IkappaB kinase 2. J Cell Sci. (2003) 116:665–74. doi: 10.1242/jcs.00286 12538767

[48] Delprat V Tellier C Demazy C Raes M Feron O Michiels C. Cycling hypoxia promotes a pro-inflammatory phenotype in macrophages via JNK/p65 signaling pathway. Sci Rep. (2020) 10:882. doi: 10.1038/s41598-020-57677-5 31964911 PMC6972721

[49] Biswas SK Lewis CE. NF-κB as a central regulator of macrophage function in tumors. J Leukoc Biol. (2010) 88:877–84. doi: 10.1189/jlb.0310153 20573802

[50] Balkwill F Charles KA Mantovani A. Smoldering and polarized inflammation in the initiation and promotion of Malignant disease. Cancer Cell. (2005) 7:211–7. doi: 10.1016/j.ccr.2005.02.013 15766659

[51] Voron T Colussi O Marcheteau E Pernot S Nizard M Pointet A-L. VEGF-A modulates expression of inhibitory checkpoints on CD8+ T cells in tumors. J Exp Med. (2015) 212:139–48. doi: 10.1084/jem.20140559 PMC4322048 25601652

[52] Poh AR Ernst M. Targeting macrophages in cancer: from bench to bedside. Front Oncol. (2018) 8:49. doi: 10.3389/fonc.2018.00049 29594035 PMC5858529

[53] Umezu D Okada N Sakoda Y Adachi K Ojima T Yamaue H. Inhibitory functions of PD-L1 and PD-L2 in the regulation of anti-tumor immunity in murine tumor microenvironment. Cancer Immunol Immunother CII. (2018) 68:201–11. doi: 10.1007/s00262-018-2263-4 PMC11028338 30357491

[54] Pittet MJ Di Pilato M Garris C Mempel TR. Dendritic cells as shepherds of T cell immunity in cancer. Immunity. (2023) 56:2218–30. doi: 10.1016/j.immuni.2023.08.014 PMC10591862 37708889

[55] Ma Y Shurin GV Peiyuan Z Shurin MR. Dendritic cells in the cancer microenvironment. J Cancer. (2013) 4:36–44. doi: 10.7150/jca.5046 23386903 PMC3564245

[56] Maier B Leader AM Chen ST Tung N Chang C LeBerichel J. A conserved dendritic-cell regulatory program limits antitumour immunity. Nature. (2020) 580:257–62. doi: 10.1038/s41586-020-2134-y PMC7787191 32269339

[57] Yoshimura S Bondeson J Brennan FM Foxwell BM Feldmann M. Role of NFkappaB in antigen presentation and development of regulatory T cells elucidated by treatment of dendritic cells with the proteasome inhibitor PSI. Eur J Immunol. (2001) 31:1883–93. doi: 10.1002/1521-4141(200106)31:6<1883::AID-IMMU1883>3.0.CO;2-V 11433385

[58] Karyampudi L Lamichhane P Krempski J Kalli KR Behrens MD Vargas DM. PD-1 Blunts the Function of Ovarian Tumor-Infiltrating Dendritic Cells by Inactivating NF-κB. Cancer Res. (2016) 76:239–50. doi: 10.1158/0008-5472.CAN-15-0748 PMC4715980 26567141

[59] Yoshimura S Bondeson J Foxwell BM Brennan FM Feldmann M. Effective antigen presentation by dendritic cells is NF-kappaB dependent: coordinate regulation of MHC, co-stimulatory molecules and cytokines. Int Immunol. (2001) 13:675–83. doi: 10.1093/intimm/13.5.675 11312255

[60] Li R Fang F Jiang M Wang C Ma J Kang W. STAT3 and NF-κB are simultaneously suppressed in dendritic cells in lung cancer. Sci Rep. (2017) 7:45395. doi: 10.1038/srep45395 28350008 PMC5368983

[61] Ghislat G Cheema AS Baudoin E Verthuy C Ballester PJ Crozat K. NF-κB-dependent IRF1 activation programs cDC1 dendritic cells to drive antitumor immunity. Sci Immunol. (2021) 6:eabg3570. doi: 10.1126/sciimmunol.abg3570 34244313

[62] Wu Y Yi M Niu M Mei Q Wu K. Myeloid-derived suppressor cells: an emerging target for anticancer immunotherapy. Mol Cancer. (2022) 21:184. doi: 10.1186/s12943-022-01657-y 36163047 PMC9513992

[63] Ahn G-O Brown JM. Matrix metalloproteinase-9 is required for tumor vasculogenesis but not for angiogenesis: role of bone marrow-derived myelomonocytic cells. Cancer Cell. (2008) 13:193–205. doi: 10.1016/j.ccr.2007.11.032 18328424 PMC2967441

[64] Li T Li X Zamani A Wang W Lee C-N Li M. c-rel is a myeloid checkpoint for cancer immunotherapy. Nat Cancer. (2020) 1:507–17. doi: 10.1038/s43018-020-0061-3 PMC7808269 33458695

[65] Tu S Bhagat G Cui G Takaishi S Kurt-Jones EA Rickman B. Overexpression of interleukin-1beta induces gastric inflammation and cancer and mobilizes myeloid-derived suppressor cells in mice. Cancer Cell. (2008) 14:408–19. doi: 10.1016/j.ccr.2008.10.011 PMC2586894 18977329

[66] Cheng P Corzo CA Luetteke N Yu B Nagaraj S Bui MM. Inhibition of dendritic cell differentiation and accumulation of myeloid-derived suppressor cells in cancer is regulated by S100A9 protein. J Exp Med. (2008) 205:2235–49. doi: 10.1084/jem.20080132 PMC2556797 18809714

[67] Kalluri R. The biology and function of fibroblasts in cancer. Nat Rev Cancer. (2016) 16:582–98. doi: 10.1038/nrc.2016.73 27550820

[68] Xiao Y Yu D. Tumor microenvironment as a therapeutic target in cancer. Pharmacol Ther. (2021) 221:107753. doi: 10.1016/j.pharmthera.2020.107753 33259885 PMC8084948

[69] Hoesel B Schmid JA. The complexity of NF-κB signaling in inflammation and cancer. Mol Cancer. (2013) 12:86. doi: 10.1186/1476-4598-12-86 23915189 PMC3750319

[70] Erez N Truitt M Olson P Arron ST Hanahan D. Cancer-associated fibroblasts are activated in incipient neoplasia to orchestrate tumor-promoting inflammation in an NF-kappaB-dependent manner. Cancer Cell. (2010) 17:135–47. doi: 10.1016/j.ccr.2009.12.041 20138012

[71] Jung D-W Che ZM Kim J Kim K Kim KY Williams D. Tumor-stromal crosstalk in invasion of oral squamous cell carcinoma: a pivotal role of CCL7. Int J Cancer. (2010) 127:332–44. doi: 10.1002/ijc.v127:2 19937793

[72] Pallangyo CK Ziegler PK Greten FR. IKKβ acts as a tumor suppressor in cancer-associated fibroblasts during intestinal tumorigenesis. J Exp Med. (2015) 212:2253–66. doi: 10.1084/jem.20150576 PMC4689166 26621452

[73] Koliaraki V Pasparakis M Kollias G. IKKβ in intestinal mesenchymal cells promotes initiation of colitis-associated cancer. J Exp Med. (2015) 212:2235–51. doi: 10.1084/jem.20150542 PMC4683996 26621453

[74] Koliaraki V Pallangyo CK Greten FR Kollias G. Mesenchymal cells in colon cancer. Gastroenterology. (2017) 152:964–79. doi: 10.1053/j.gastro.2016.11.049 28111227

[75] Schwitalla S Fingerle AA Cammareri P Nebelsiek T Göktuna SI Ziegler PK. Intestinal tumorigenesis initiated by dedifferentiation and acquisition of stem-cell-like properties. Cell. (2013) 152:25–38. doi: 10.1016/j.cell.2012.12.012 23273993

[76] Bollrath J Phesse TJ von Burstin VA Putoczki T Bennecke M Bateman T. gp130-mediated Stat3 activation in enterocytes regulates cell survival and cell-cycle progression during colitis-associated tumorigenesis. Cancer Cell. (2009) 15:91–102. doi: 10.1016/j.ccr.2009.01.002 19185844

[77] Oh A Pardo M Rodriguez A Yu C Nguyen L Liang O. NF-κB signaling in neoplastic transition from epithelial to mesenchymal phenotype. Cell Commun Signal CCS. (2023) 21:291. doi: 10.1186/s12964-023-01207-z 37853467 PMC10585759

[78] Pires BRB Mencalha AL Ferreira GM de Souza WF Morgado-Díaz JA Maia AM. NF-kappaB is involved in the regulation of EMT genes in breast cancer cells. PloS One. (2017) 12:e0169622. doi: 10.1371/journal.pone.0169622 28107418 PMC5249109

[79] Yu H Pardoll D Jove R. STATs in cancer inflammation and immunity: a leading role for STAT3. Nat Rev Cancer. (2009) 9:798–809. doi: 10.1038/nrc2734 19851315 PMC4856025

[80] Wang Y Zhou BP. Epithelial-mesenchymal transition in breast cancer progression and metastasis. Chin J Cancer. (2011) 30:603–11. doi: 10.5732/cjc.011.10226 PMC3702729 21880181

[81] Li Y He J Wang F Wang X Yang F Zhao C. Role of MMP-9 in epithelial-mesenchymal transition of thyroid cancer. World J Surg Oncol. (2020) 18:181. doi: 10.1186/s12957-020-01958-w 32698816 PMC7376963

[82] Al-Sadi R Engers J Haque M King S Al-Omari D Ma TY. Matrix Metalloproteinase-9 (MMP-9) induced disruption of intestinal epithelial tight junction barrier is mediated by NF-κB activation. PloS One. (2021) 16:e0249544. doi: 10.1371/journal.pone.0249544 33826658 PMC8026081

[83] Wang F He W Fanghui P Wang L Fan Q. NF-κBP65 promotes invasion and metastasis of oesophageal squamous cell cancer by regulating matrix metalloproteinase-9 and epithelial-to-mesenchymal transition. Cell Biol Int. (2013) 37:780–8. doi: 10.1002/cbin.10089 23504993

[84] Leone P Malerba E Susca N Favoino E Perosa F Brunori G. Endothelial cells in tumor microenvironment: insights and perspectives. Front Immunol. (2024) 15:1367875. doi: 10.3389/fimmu.2024.1367875 38426109 PMC10902062

[85] Sun H-W Li C-J Chen H-Q Lin H-L Lv H-X Zhang Y. Involvement of integrins, MAPK, and NF-kappaB in regulation of the shear stress-induced MMP-9 expression in endothelial cells. Biochem Biophys Res Commun. (2007) 353:152–8. doi: 10.1016/j.bbrc.2006.12.002 17174275

[86] Ko H-M Park Y-M Jung B Kim H-A Choi J-H Park SJ. Involvement of matrix metalloproteinase-9 in platelet-activating factor-induced angiogenesis. FEBS Lett. (2005) 579:2369–75. doi: 10.1016/j.febslet.2005.03.035 15848174

[87] Ferrara N Gerber H-P LeCouter J. The biology of VEGF and its receptors. Nat Med. (2003) 9:669–76. doi: 10.1038/nm0603-669 12778165

[88] Kolch W Martiny-Baron G Kieser A Marmé D. Regulation of the expression of the VEGF/VPS and its receptors: role in tumor angiogenesis. Breast Cancer Res Treat. (1995) 36:139—155. doi: 10.1007/BF00666036 8534863

[89] Dong F Zhou X Li C Yan S Deng X Cao Z. Dihydroartemisinin targets VEGFR2 via the NF-κB pathway in endothelial cells to inhibit angiogenesis. Cancer Biol Ther. (2014) 15:1479–88. doi: 10.4161/15384047.2014.955728 PMC4623302 25482945

[90] Meng W Xue S Chen Y. The role of CXCL12 in tumor microenvironment. Gene. (2018) 641:105–10. doi: 10.1016/j.gene.2017.10.015 29017963

[91] Madge LA Kluger MS Orange JS May MJ. Lymphotoxin-alpha 1 beta 2 and LIGHT induce classical and noncanonical NF-kappa B-dependent proinflammatory gene expression in vascular endothelial cells. J Immunol Baltim. Md 1950. (2008) 180:3467–77. doi: 10.4049/jimmunol.180.5.3467 PMC2596750 18292573

[92] Noort AR van Zoest KP Weijers EM Koolwijk P Maracle CX Novack DV. NF-κB-inducing kinase is a key regulator of inflammation-induced and tumour-associated angiogenesis. J Pathol. (2014) 234:375–85. doi: 10.1002/path.4403 PMC4194146 25043127

[93] Teicher BA Fricker SP. CXCL12 (SDF-1)/CXCR4 pathway in cancer. Clin Cancer Res. (2010) 16:2927–31. doi: 10.1158/1078-0432.CCR-09-2329 20484021

[94] Martin T Cardarelli PM Parry GC Felts KA Cobb RR. Cytokine induction of monocyte chemoattractant protein-1 gene expression in human endothelial cells depends on the cooperative action of NF-kappa B and AP-1. Eur J Immunol. (1997) 27:1091–7. doi: 10.1002/eji.1830270508 9174597

[95] Vento-Tormo R Rodríguez-Ubreva J Lisio LD Islam ABMMK Urquiza JM Hernando H. NF-κB directly mediates epigenetic deregulation of common microRNAs in Epstein-Barr virus-mediated transformation of B-cells and in lymphomas. Nucleic Acids Res. (2014) 42:11025–39. doi: 10.1093/nar/gku826 PMC4176189 25200074

[96] Ghosh A Saginc G Leow SC Khattar E Shin EM Yan TD. Telomerase directly regulates NF-κB-dependent transcription. Nat Cell Biol. (2012) 14:1270–81. doi: 10.1038/ncb2621 23159929

[97] Karin M Lin A. NF-kappaB at the crossroads of life and death. Nat Immunol. (2002) 3:221–7. doi: 10.1038/ni0302-221 11875461

[98] Huang S Pettaway CA Uehara H Bucana CD Fidler IJ. Blockade of NF-kappaB activity in human prostate cancer cells is associated with suppression of angiogenesis, invasion, and metastasis. Oncogene. (2001) 20:4188–97. doi: 10.1038/sj.onc.1204535 11464285

[99] Bergers G Brekken R McMahon G Vu TH Itoh T Tamaki K. Matrix metalloproteinase-9 triggers the angiogenic switch during carcinogenesis. Nat Cell Biol. (2000) 2:737–44. doi: 10.1038/35036374 PMC2852586 11025665

[100] Song ZB Ni J-S Wu P Bao YL Liu T Li M. Testes-specific protease 50 promotes cell invasion and metastasis by increasing NF-kappaB-dependent matrix metalloproteinase-9 expression. Cell Death Dis. (2015) 6:e1703. doi: 10.1038/cddis.2015.61 25811800 PMC4385939

[101] Levine L Lucci JA Pazdrak B Cheng J-Z Guo Y-S Townsend CM. Bombesin stimulates nuclear factor kappa B activation and expression of proangiogenic factors in prostate cancer cells. Cancer Res. (2003) 63:3495–502. doi: 10.4049/jimmunol.153.5.2052 12839933

[102] Lennikov A Mirabelli P Mukwaya A Schaupper M Thangavelu M Lachota M. Selective IKK2 inhibitor IMD0354 disrupts NF-κB signaling to suppress corneal inflammation and angiogenesis. Angiogenesis. (2018) 21:267–85. doi: 10.1007/s10456-018-9594-9 PMC5878206 29332242

[103] Zhai B-T Tian H Sun J Zou J-B Zhang X-F Cheng J-X. Urokinase-type plasminogen activator receptor (uPAR) as a therapeutic target in cancer. J Transl Med. (2022) 20:135. doi: 10.1186/s12967-022-03329-3 35303878 PMC8932206

[104] Wang W Abbruzzese J Evans D Chiao P. Overexpression of urokinase-type plasminogen activator in pancreatic adenocarcinoma is regulated by constitutively activated RelA. Oncogene. (1999) 18:4554—4563. doi: 10.1038/sj.onc.1202833 10467400

[105] Sliva D English D Lyons D Lloyd FP. Protein kinase C induces motility of breast cancers by upregulating secretion of urokinase-type plasminogen activator through activation of AP-1 and NF-kappaB. Biochem Biophys Res Commun. (2002) 290:552–7. doi: 10.1006/bbrc.2001.6225 11779207

[106] Garris CS Arlauckas SP Kohler RH Trefny MP Garren S Piot C. Successful anti-PD-1 cancer immunotherapy requires T cell-dendritic cell crosstalk involving the cytokines IFN-γ and IL-12. Immunity. (2018) 49:1148–1161.e7. doi: 10.1016/j.immuni.2018.09.024 30552023 PMC6301092

[107] Hoffmann E Dittrich-Breiholz O Holtmann H Kracht M. Multiple control of interleukin-8 gene expression. J Leukoc Biol. (2002) 72:847–55. doi: 10.1189/jlb.72.5.847 12429706

[108] Bruni-Cardoso A Johnson LC Vessella RL Peterson TE Lynch CC. Osteoclast-derived matrix metalloproteinase-9 directly affects angiogenesis in the prostate tumor-bone microenvironment. Mol Cancer Res MCR. (2010) 8:459–70. doi: 10.1158/1541-7786.MCR-09-0445 PMC2946627 20332212

[109] Ueda A Okuda K Ohno S Shirai A Igarashi T Matsunaga K. NF-kappa B and Sp1 regulate transcription of the human monocyte chemoattractant protein-1 gene. J Immunol Baltim. Md 1950. (1994) 153:2052—2063. doi: 10.4049/jimmunol.153.5.2052 8051410

[110] Passaro C Borriello F Vastolo V Di Somma S Scamardella E Gigantino V. The oncolytic virus dl922-947 reduces IL-8/CXCL8 and MCP-1/CCL2 expression and impairs angiogenesis and macrophage infiltration in anaplastic thyroid carcinoma. Oncotarget. (2016) 7:1500–15. doi: 10.18632/oncotarget.6430 PMC4811476 26625205

[111] Greten FR Eckmann L Greten TF Park JM Li Z-W Egan LJ. IKKbeta links inflammation and tumorigenesis in a mouse model of colitis-associated cancer. Cell. (2004) 118:285–96. doi: 10.1016/j.cell.2004.07.013 15294155

[112] Grivennikov S Karin E Terzic J Mucida D Yu G-Y Vallabhapurapu S. IL-6 and Stat3 are required for survival of intestinal epithelial cells and development of colitis-associated cancer. Cancer Cell. (2009) 15:103—113. doi: 10.1016/j.ccr.2009.01.001 19185845 PMC2667107

[113] Müerköster S Wegehenkel K Arlt A Witt M Sipos B Kruse M-L. Tumor stroma interactions induce chemoresistance in pancreatic ductal carcinoma cells involving increased secretion and paracrine effects of nitric oxide and interleukin-1beta. Cancer Res. (2004) 64:1331–7. doi: 10.1158/0008-5472.can-03-1860 14973050

[114] Benedyk M Sopalla C Nacken W Bode G Melkonyan H Banfi B. HaCaT keratinocytes overexpressing the S100 proteins S100A8 and S100A9 show increased NADPH oxidase and NF-kappaB activities. J Invest Dermatol. (2007) 127:2001–11. doi: 10.1038/sj.jid.5700820 17429438

[115] Grivennikov SI Karin M. Dangerous liaisons: STAT3 and NF-kappaB collaboration and crosstalk in cancer. Cytokine Growth Factor Rev. (2010) 21:11–9. doi: 10.1016/j.cytogfr.2009.11.005 PMC2834864 20018552

[116] Mao X Xu J Wang W Liang C Hua J Liu J. Crosstalk between cancer-associated fibroblasts and immune cells in the tumor microenvironment: new findings and future perspectives. Mol Cancer. (2021) 20:131. doi: 10.1186/s12943-021-01428-1 34635121 PMC8504100

[117] Katakam AK Brightbill H Franci C Kung C Nunez V Jones C. Dendritic cells require NIK for CD40-dependent cross-priming of CD8+ T cells. Proc Natl Acad Sci U S A. (2015) 112:14664–9. doi: 10.1073/pnas.1520627112 PMC4664370 26561586

[118] Rahma OE Hodi FS. The intersection between tumor angiogenesis and immune suppression. Clin Cancer Res Off J Am Assoc Cancer Res. (2019) 25:5449–57. doi: 10.1158/1078-0432.CCR-18-1543 30944124

[119] Balkwill F Mantovani A. Inflammation and cancer: back to Virchow? Lancet Lond Engl. (2001) 357:539–45. doi: 10.1016/S0140-6736(00)04046-0 11229684

[120] Pikarsky E Porat RM Stein I Abramovitch R Amit S Kasem S. NF-kappaB functions as a tumour promoter in inflammation-associated cancer. Nature. (2004) 431:461–6. doi: 10.1038/nature02924 15329734

[121] Kishimoto T. Interleukin-6: from basic science to medicine–40 years in immunology. Annu Rev Immunol. (2005) 23:1–21. doi: 10.1146/annurev.immunol.23.021704.115806 15771564

[122] Disis ML. Immune regulation of cancer. J Clin Oncol Off J Am Soc Clin Oncol. (2010) 28:4531–8. doi: 10.1200/JCO.2009.27.2146 PMC3041789 20516428

[123] Dajee M Lazarov M Zhang JY Cai T Green CL Russell AJ. NF-kappaB blockade and oncogenic Ras trigger invasive human epidermal neoplasia. Nature. (2003) 421:639–43. doi: 10.1038/nature01283 12571598

[124] van Hogerlinden M Rozell BL Ahrlund-Richter L Toftgård R. Squamous cell carcinomas and increased apoptosis in skin with inhibited Rel/nuclear factor-kappaB signaling. Cancer Res. (1999) 59:3299–303. 10416581

[125] Yang J Hawkins OE Barham W Gilchuk P Boothby M Ayers GD. Myeloid IKKβ promotes antitumor immunity by modulating CCL11 and the innate immune response. Cancer Res. (2014) 74:7274–84. doi: 10.1158/0008-5472.CAN-14-1091 PMC4349570 25336190

[126] Greten FR Eckmann L Greten TF Park JM Li Z-W Egan LJ. IKKβ Links inflammation and tumorigenesis in a mouse model of colitis-associated cancer. Cell. (2004) 118:285–96. doi: 10.1016/j.cell.2004.07.013 15294155

[127] Luedde T Beraza N Kotsikoris V van Loo G Nenci A De Vos R. Deletion of NEMO/IKKgamma in liver parenchymal cells causes steatohepatitis and hepatocellular carcinoma. Cancer Cell. (2007) 11:119–32. doi: 10.1016/j.ccr.2006.12.016 17292824

[128] Yu H Kortylewski M Pardoll D. Crosstalk between cancer and immune cells: role of STAT3 in the tumour microenvironment. Nat Rev Immunol. (2007) 7:41–51. doi: 10.1038/nri1995 17186030

[129] Yu H Jove R. The STATs of cancer–new molecular targets come of age. Nat Rev Cancer. (2004) 4:97–105. doi: 10.1038/nrc1275 14964307

[130] Yang J Liao X Agarwal MK Barnes L Auron PE Stark GR. Unphosphorylated STAT3 accumulates in response to IL-6 and activates transcription by binding to NFkappaB. Genes Dev. (2007) 21:1396–408. doi: 10.1101/gad.1553707 PMC1877751 17510282

[131] Kortylewski M Kujawski M Wang T Wei S Zhang S Pilon-Thomas S. Inhibiting Stat3 signaling in the hematopoietic system elicits multicomponent antitumor immunity. Nat Med. (2005) 11:1314–21. doi: 10.1038/nm1325 16288283

[132] Wang T Niu G Kortylewski M Burdelya L Shain K Zhang S. Regulation of the innate and adaptive immune responses by Stat-3 signaling in tumor cells. Nat Med. (2004) 10:48–54. doi: 10.1038/nm976 14702634

[133] He G Yu G-Y Temkin V Ogata H Kuntzen C Sakurai T. Hepatocyte IKKbeta/NF-kappaB inhibits tumor promotion and progression by preventing oxidative stress-driven STAT3 activation. Cancer Cell. (2010) 17:286–97. doi: 10.1016/j.ccr.2009.12.048 PMC2841312 20227042

[134] Zhang X Wang L Qu Y. Targeting the β-catenin signaling for cancer therapy. Pharmacol Res. (2020) 160:104794. doi: 10.1016/j.phrs.2020.104794 32278038

[135] Zhang Y Wang X. Targeting the Wnt/β-catenin signaling pathway in cancer. J Hematol Oncol.J Hematol Oncol. (2020) 13:165. doi: 10.1186/s13045-020-00990-3 33276800 PMC7716495

[136] Bienz M Clevers H. Linking colorectal cancer to Wnt signaling. Cell. (2000) 103:311–20. doi: 10.1016/S0092-8674(00)00122-7 11057903

[137] Zhao H Ming T Tang S Ren S Yang H Liu M. Wnt signaling in colorectal cancer: pathogenic role and therapeutic target. Mol Cancer. (2022) 21:144. doi: 10.1186/s12943-022-01616-7 35836256 PMC9281132

[138] Halma MTJ Tuszynski JA Marik PE. Cancer metabolism as a therapeutic target and review of interventions. Nutrients. (2023) 15:4245. doi: 10.3390/nu15194245 37836529 PMC10574675

[139] Chelakkot C Chelakkot VS Shin Y Song K. Modulating glycolysis to improve cancer therapy. Int J Mol Sci. (2023) 24:2606. doi: 10.3390/ijms24032606 36768924 PMC9916680

[140] Kawauchi K Araki K Tobiume K Tanaka N. p53 regulates glucose metabolism through an IKK-NF-kappaB pathway and inhibits cell transformation. Nat Cell Biol. (2008) 10:611–8. doi: 10.1038/ncb1724 18391940

[141] Reid MA Lowman XH Pan M Tran TQ Warmoes MO Ishak Gabra MB. IKKβ promotes metabolic adaptation to glutamine deprivation via phosphorylation and inhibition of PFKFB3. Genes Dev. (2016) 30:1837–51. doi: 10.1101/gad.287235.116 PMC5024682 27585591

[142] Bian X Liu R Meng Y Xing D Xu D Lu Z. Lipid metabolism and cancer. J Exp Med. (2021) 218:e20201606. doi: 10.1084/jem.20201606 33601415 PMC7754673

[143] Jiang M Wu N Xu B Chu Y Li X Su S. Fatty acid-induced CD36 expression via O-GlcNAcylation drives gastric cancer metastasis. Theranostics. (2019) 9:5359–73. doi: 10.7150/thno.34024 PMC6691574 31410220

[144] Capece D D’Andrea D Begalli F Goracci L Tornatore L Alexander JL. Enhanced triacylglycerol catabolism by carboxylesterase 1 promotes aggressive colorectal carcinoma. J Clin Invest. (2021) 131:e137845. doi: 10.1172/JCI137845 33878036 PMC8159693

[145] Molaei M Vandehoef C Karpac J. NF-κB shapes metabolic adaptation by attenuating foxo-mediated lipolysis in drosophila. Dev Cell. (2019) 49:802–810.e6. doi: 10.1016/j.devcel.2019.04.009 31080057 PMC6548632

[146] Fuhrmeister J Zota A Sijmonsma TP Seibert O Cıngır Ş Schmidt K. Fasting-induced liver GADD45β restrains hepatic fatty acid uptake and improves metabolic health. EMBO Mol Med. (2016) 8:654–69. doi: 10.15252/emmm.201505801 PMC4888855 27137487

[147] Verzella D Bennett J Fischietti M Thotakura AK Recordati C Pasqualini F. GADD45β Loss ablates innate immunosuppression in cancer. Cancer Res. (2018) 78:1275–92. doi: 10.1158/0008-5472.CAN-17-1833 PMC5935595 29279355

[148] Moretti M Bennett J Tornatore L Thotakura AK Franzoso G. Cancer: NF-κB regulates energy metabolism. Int J Biochem Cell Biol. (2012) 44:2238–43. doi: 10.1016/j.biocel.2012.08.002 22903018

[149] Mauro C Leow SC Anso E Rocha S Thotakura AK Tornatore L. NF-κB controls energy homeostasis and metabolic adaptation by upregulating mitochondrial respiration. Nat Cell Biol. (2011) 13:1272–9. doi: 10.1038/ncb2324 PMC3462316 21968997

[150] Loftus RM Finlay DK. Immunometabolism: cellular metabolism turns immune regulator. J Biol Chem. (2016) 291:1–10. doi: 10.1074/jbc.R115.693903 26534957 PMC4697146

[151] Wu D Tian S Zhu W. Modulating multidrug resistance to drug-based antitumor therapies through NF-κB signaling pathway: mechanisms and perspectives. Expert Opin Ther Targets. (2023) 27:503–15. doi: 10.1080/14728222.2023.2225767 37314372

[152] Wilson AJ Barham W Saskowski J Tikhomirov O Chen L Lee H-J. Tracking NF-κB activity in tumor cells during ovarian cancer progression in a syngeneic mouse model. J Ovarian Res. (2013) 6:63. doi: 10.1186/1757-2215-6-63 24020521 PMC3846584

[153] Smith MP Sanchez-Laorden B O’Brien K Brunton H Ferguson J Young H. The immune microenvironment confers resistance to MAPK pathway inhibitors through macrophage-derived TNFα. Cancer Discovery. (2014) 4:1214–29. doi: 10.1158/2159-8290.CD-13-1007 PMC4184867 25256614

[154] Liu J Lao L Chen J Li J Zeng W Zhu X. The IRENA lncRNA converts chemotherapy-polarized tumor-suppressing macrophages to tumor-promoting phenotypes in breast cancer. Nat Cancer. (2021) 2:457–73. doi: 10.1038/s43018-021-00196-7 35122000

[155] Yang Y-I Wang Y-Y Ahn J-H Kim B-H Choi J-H. CCL2 overexpression is associated with paclitaxel resistance in ovarian cancer cells via autocrine signaling and macrophage recruitment. Biomed Pharmacother. (2022) 153:113474. doi: 10.1016/j.biopha.2022.113474 36076499

[156] Le F Yang L Han Y Zhong Y Zhan F Feng Y. TPL inhibits the invasion and migration of drug-resistant ovarian cancer by targeting the PI3K/AKT/NF-κB-signaling pathway to inhibit the polarization of M2 TAMs. Front Oncol. (2021) 11:704001. doi: 10.3389/fonc.2021.704001 34381726 PMC8350572

[157] Yang T Deng Z Xu L Li X Yang T Qian Y. Macrophages-aPKCι-CCL5 feedback loop modulates the progression and chemoresistance in cholangiocarcinoma. J Exp Clin Cancer Res CR. (2022) 41:23. doi: 10.1186/s13046-021-02235-8 35033156 PMC8760815

[158] Zhou Y Tang W Zhuo H Zhu D Rong D Sun J. Cancer-associated fibroblast exosomes promote chemoresistance to cisplatin in hepatocellular carcinoma through circZFR targeting signal transducers and activators of transcription (STAT3)/nuclear factor -kappa B (NF-κB) pathway. Bioengineered. (2022) 13:4786–97. doi: 10.1080/21655979.2022.2032972 PMC8973934 35139763

[159] Zhang X Zheng S Hu C Li G Lin H Xia R. Cancer-associated fibroblast-induced lncRNA UPK1A-AS1 confers platinum resistance in pancreatic cancer via efficient double-strand break repair. Oncogene. (2022) 41:2372–89. doi: 10.1038/s41388-022-02253-6 PMC9010302 35264742

[160] Zhai J Shen J Xie G Wu J He M Gao L. Cancer-associated fibroblasts-derived IL-8 mediates resistance to cisplatin in human gastric cancer. Cancer Lett. (2019) 454:37–43. doi: 10.1016/j.canlet.2019.04.002 30978440

[161] Liu M Wei F Wang J Yu W Shen M Liu T. Myeloid-derived suppressor cells regulate the immunosuppressive functions of PD-1-PD-L1+ Bregs through PD-L1/PI3K/AKT/NF-κB axis in breast cancer. Cell Death Dis. (2021) 12:465. doi: 10.1038/s41419-021-03745-1 33967272 PMC8107179

[162] Di Pilato M Kim EY Cadilha BL Prüßmann JN Nasrallah MN Seruggia D. Targeting the CBM complex causes Treg cells to prime tumours for immune checkpoint therapy. Nature. (2019) 570:112–6. doi: 10.1038/s41586-019-1215-2 PMC6656391 31092922

[163] Prescott JA Cook SJ. Targeting IKKβ in cancer: challenges and opportunities for the therapeutic utilisation of IKKβ Inhibitors. Cells. (2018) 7:115. doi: 10.3390/cells7090115 30142927 PMC6162708

[164] Mantovani A Sica A. Macrophages, innate immunity and cancer: balance, tolerance, and diversity. Curr Opin Immunol. (2010) 22:231–7. doi: 10.1016/j.coi.2010.01.009 20144856

[165] Mantovani A Sozzani S Locati M Allavena P Sica A. Macrophage polarization: tumor-associated macrophages as a paradigm for polarized M2 mononuclear phagocytes. Trends Immunol. (2002) 23:549–55. doi: 10.1016/S1471-4906(02)02302-5 12401408

[166] Martin M Wei H Lu T. Targeting microenvironment in cancer therapeutics. Oncotarget. (2016) 7:52575–83. doi: 10.18632/oncotarget.v7i32 PMC5239574 27270649

[167] Compagno M Lim WK Grunn A Nandula SV Brahmachary M Shen Q. Mutations of multiple genes cause deregulation of NF-kappaB in diffuse large B-cell lymphoma. Nature. (2009) 459:717–21. doi: 10.1038/nature07968 PMC2973325 19412164

